# Accurate Spatial Heterogeneity Dissection and Gene Regulation Interpretation for Spatial Transcriptomics using Dual Graph Contrastive Learning

**DOI:** 10.1002/advs.202410081

**Published:** 2024-11-28

**Authors:** Zhuohan Yu, Yuning Yang, Xingjian Chen, Ka‐Chun Wong, Zhaolei Zhang, Yuming Zhao, Xiangtao Li

**Affiliations:** ^1^ School of Artificial Intelligence Jilin University Jilin 130012 China; ^2^ Terrence Donnelly Centre for Cellular and Biomolecular Research University of Toronto Toronto ON M5S 3E1 Canada; ^3^ Cutaneous Biology Research Center, Massachusetts General Hospital Harvard Medical School Boston MA 02115 USA; ^4^ Department of Computer Science City University of Hong Kong Hong Kong SAR 999077 Hong Kong; ^5^ College of Computer and Control Engineering Northeast Forestry University Harbin 150040 China

**Keywords:** dual graph contrastive learning, gene regulation, graph contrastive learning, spatial heterogeneity

## Abstract

Recent advances in spatial transcriptomics have enabled simultaneous preservation of high‐throughput gene expression profiles and the spatial context, enabling high‐resolution exploration of distinct regional characterization in tissue. To effectively understand the underlying biological mechanisms within tissue microenvironments, there is a requisite for methods that can accurately capture external spatial heterogeneity and interpret internal gene regulation from spatial transcriptomics data. However, current methods for region identification often lack the simultaneous characterizing of spatial structure and gene regulation, thereby limiting the ability of spatial dissection and gene interpretation. Here, stDCL is developed, a dual graph contrastive learning method to identify spatial domains and interpret gene regulation in spatial transcriptomics data. stDCL adaptively incorporates gene expression data and spatial information via a graph embedding autoencoder, thereby preserving critical information within the latent embedding representations. In addition, dual graph contrastive learning is proposed to train the model, ensuring that the latent embedding representation closely resembles the actual spatial distribution and exhibits cluster similarity. Benchmarking stDCL against other state‐of‐the‐art clustering methods using complex cortex datasets demonstrates its superior accuracy and effectiveness in identifying spatial domains. Our analysis of the imputation matrices generated by stDCL reveals its capability to reconstruct spatial hierarchical structures and refine differential expression assessment. Furthermore, it is demonstrated that the versatility of stDCL in interpretability of gene regulation, spatial heterogeneity at high resolution, and embryonic developmental patterns. In addition, it is also showed that stDCL can successfully annotate disease‐associated astrocyte subtypes in Alzheimer's disease and unravel multiple relevant pathways and regulatory mechanisms.

## Graphical abstract

stDCL, a dual graph contrastive learning method, captures spatial heterogeneity and interprets gene regulation in spatial transcriptomics data. Integrating spatial and gene expression data through graph embeddings, stDCL provides robust spatial characterization and accurate region identification, reconstructs spatial hierarchies, and identifies disease‐associated cell subtypes, unveiling new insights into tissue microenvironments and disease mechanisms.

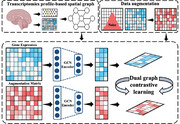

## 1 Introduction

Spatial transcriptomics involves a series of emerging genomic technologies that reveal gene expression in tissues with spatial context, thereby providing a deeper understanding of the structure and function of tissues at the spatial level.^[^ ^1^ ^]^ Several spatial transcriptomics technologies have been developed, including RNA sequencing‐based technologies such as 10x Visium,^[^ ^2^ ^]^ Slide‐seq,^[^ ^3^ ^]^ and spatial transcriptomics (ST),^[^ ^4^ ^]^ which enable genome‐wide analysis of gene expression by thousands across tissue locations. In addition, single‐molecule fluorescence in situ hybridization (smFISH)‐based technologies, such as MERFISH,^[^ ^5^, ^6^, ^7^ ^]^ seqFISH,^[^ ^8^, ^9^ ^]^ and seqFISH plus,^[^ ^10^ ^]^ offer single‐cell resolution, bridging gene expression and spatial position. These advancements allow studying spatial gene expression patterns,^[^ ^11^, ^12^ ^]^ exploring the transcriptomic landscape of tissues, and inferring cell‐to‐cell communication.^[^ ^13^ ^]^ In general, spatial transcriptomics has facilitated new discoveries in many areas of biology.^[^ ^14^, ^15^ ^]^

The spatial heterogeneity of complex tissue architecture is fundamentally related to the spatial domains of different regions. These domains, characterized by their distinct gene expression patterns and histological characteristics, underlie various biological functions and regulatory mechanisms. They are characterized by distinct cellular compositions, transcriptomic diversity, and cell‐to‐cell interactions, all of which are critical to understanding physiology and pathology.^[^ ^16^, ^17^, ^18^ ^]^ Therefore, it is crucial to identify and determine the spatial domains in tissues. Traditional clustering algorithms, such as K‐means and Louvain, rely primarily on gene expression data and ignore critical spatial information, resulting in clustering results that fail to accurately represent the intricacies of the actual tissue. To mitigate this issue, several methodologies incorporating spatial information for enhanced identification of spatial domains have been developed. Among these, stLearn^[^ ^19^ ^]^ extracts morphological features from morphological images and spatial locations using deep learning models and normalizes gene expression using morphological distances. BayesSpace,^[^ ^20^ ^]^ a Bayesian statistical clustering method, imposes a prior for ST data and encourages spatially adjacent spots to belong to the same cluster. SpaGCN^[^ ^21^ ^]^ employs graph convolution networks to integrate gene expression with weighted graphs constructed from spatial distance and histology to identify spatial domains. STAGATE^[^ ^22^ ^]^ develops an adaptive graph attention autoencoder to learn low‐dimensional latent embeddings of ST data and identify spatial domains. CCST^[^ ^23^ ^]^ uses unsupervised GCNs to learn a cell embedding representation based on graphs extracted from spatial transcriptomics data. However, although these methods combine gene expression and spatial information to identify spatial domains, they are all based entirely on unsupervised learning, resulting in the learned embedding representations often lacking the fidelity required to accurately segment spatial domains, making it prone to generate discrepancies between identified domains and pathology annotations.

In recent years, various methods rooted in self‐supervised learning have emerged to enhance the optimization of embedding representations and elevate the efficacy of spatial domain identification. SpaceFlow^[^ ^24^ ^]^ uses a spatially regularized deep graph network to combine gene expression similarity with spatial information through a contrastive learning strategy. conST^[^ ^25^ ^]^ explores multiple patterns of ST data through a contrastive learning framework in two training stages and learns to embed representations beneficial for diverse downstream tasks. Similarly, GraphST^[^ ^26^ ^]^ introduces self‐supervised graph contrastive learning to learn informative representations of gene expression profiles alongside their spatial coordinates, thus enriching latent representation learning. ConGI^[^ ^27^ ^]^ deciphers the spatial domains by adapting gene expression to histopathology images through contrastive learning. However, these methods typically lack ability to fully utilize the spatial information within their self‐supervised contrastive learning designs, resulting in inadequate guidance of model training by spatial information, thus perpetuating the difficulty of accurately identifying spatial domains. Moreover, existing methods are unable to optimize the latent embedding representations at both spatial and gene levels, thereby ignoring many key gene regulations.

Here, we develop stDCL, a graph convolutional autoencoder framework that uses dual contrastive learning to identify spatial domains from spatial transcriptomics data. stDCL integrates spatial information and gene expression information through graph convolutional networks (GCNs) to learn the latent embedding representations and allow imputation of ST data. Then, stDCL employs dual graph contrastive learning (space‐aware contrastive learning and cluster‐level feature contrastive learning) to enable the embedding representation and imputation matrix to preserve the important spatial heterogeneity and gene regulation. The space‐aware contrastive learning improves the similarity between spots of close spatial location while reducing the similarity between spots of distant spatial location within the representation space. The cluster‐level feature contrastive learning brings the intra‐cluster spots closer and further away from the inter‐cluster spots in terms of feature dimensionality. Evaluation of stDCL's clustering performance on the human dorsolateral prefrontal cortex (DLPFC) 10x Visium dataset and frontal cortex MERFISH dataset demonstrated its superiority over eight state‐of‐the‐art methods for identifying spatial domains. In particular, stDCL exhibited robust performance even on complex tissue structures and detected substructures not detected by other methods. Additionally, we analyzed the interpretable functional genes in the latent embedding representation of stDCL and aggregated their spatial expression information to provide a reliable comprehension of multiple regions. Furthermore, the imputation of stDCL could effectively reconstruct the hierarchical structure of the spatial transcriptomics data, enhancing layer‐specific differential gene identification while mitigating noise. We also demonstrated that stDCL can reveal spatial heterogeneity between different regions at high spatial resolution and unravel patterns of brain development in mouse embryos. Moreover, stDCL proved valuable in identifying cellular phenotypes associated with Alzheimer's disease, elucidating the complex regulatory processes underlying its pathology.

## 2 Results

### 2.1 Overview of stDCL

**Figure 1:**
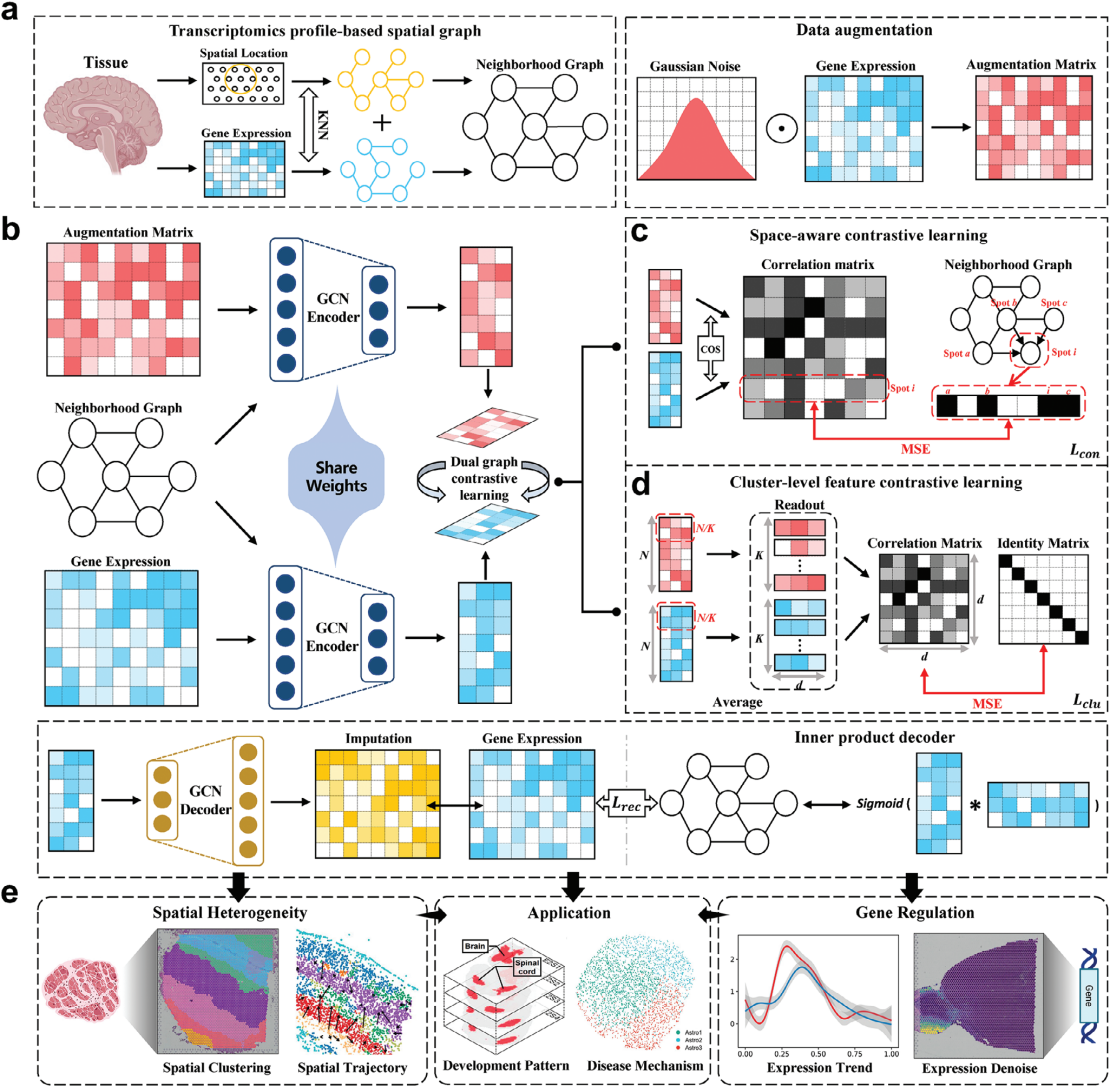
Overview of stDCL. a) The graph construction process for spatial transcriptomics data via spatial location and gene expression. b) stDCL learns the low‐dimensional latent representation and the imputation matrix with spatial information and gene expression information via the graph convolutional autoencoder. c) stDCL uses a weight‐shared GCN encoder to encode the corrupted gene expression matrix generated by data augmentation and obtains the latent embedding representations in different views. The space‐aware contrastive learning is used to further learn the relationships between spots on the latent representation. d) stDCL uses the cluster‐level feature contrastive learning to strengthen the correlation of the same dimension features in two views and improve the clustering performance of the model. e) The application capabilities of latent embedding representations trained and imputation matrices by stDCL.

stDCL learns the low‐dimensional latent embedding representation from spatial information and gene expression information by dual graph contrastive losses. As depicted in **Figure** **1**, the workflow of stDCL comprises four principal stages: (1) constructing a transcriptomics profile‐based spatial graph that merges gene expression and spatial data from spatial transcriptomics (ST) data; (2) deriving the low‐dimensional latent representations using a graph convolutional autoencoder; (3) bringing spatially neighboring spots closer and separating non‐neighboring spots via space‐aware contrastive learning; and (4) utilizing cluster‐level feature contrastive learning to augment the correlation between homologous dimensional genes based on cluster‐level representations derived from the readout function.

stDCL first constructs a transcriptomics profile‐based spatial graph by integrating gene expression information and spatial information to jointly characterize and balance the relationship between spots at gene‐specific and spatial levels (Figure 1a). Subsequently, the process continues with the use of a preprocessed gene expression matrix and the neighborhood graph as inputs into graph convolutional networks (GCNs), serving both as encoder and decoder to effectively preserve the underlying topological structure inherent in the latent embedding representations (Figure 1b). At the end, through optimizing the expression matrix and the loss function associated with graph reconstruction, stDCL learns to derive low‐dimensional latent embedding representations and imputation matrices, leveraging the spatial and gene expression information.

In addition, we propose a dual graph contrastive learning model in stDCL to learn graph‐level discriminative representations efficiently. We first generate a corrupted gene expression matrix by adding Gaussian noise to the original gene expression matrix to obtain different views of genetic characterization for data augmentation. Then, stDCL feeds the corrupted gene expression matrix into the shared encoder, yielding cross‐view latent characterizations. Further, to preserve the underlying spatial similarities and topological relationships between cellular spots, we propose a space‐aware contrastive learning approach that harnesses spatial information to ensure alignment between the spots in the embedding representations and their true spatial distribution (Figure 1c). Specifically, we calculate the mean squared error (MSE) of the cosine similarity matrix derived from the cross‐view representations utilizing the neighborhood graph, effectively assigning higher similarity scores to spots in close spatial proximity and lower scores to those in distant spatial locations during the training process. On this basis, we also propose a cluster‐level feature contrastive learning strategy to enhance the spatial clustering proficiency of our model (Figure 1d), which utilizes a readout function to calculate cluster‐level embedding representations, achieved by averaging the cross‐view representations for each cluster. Subsequently, we compute the mean squared error (MSE) between the identity matrix and the cosine similarity matrix of the cluster‐level embedding representations to draw representations of the identical dimensional features closer together. Finally, the cluster‐level feature contrastive learning approach is proposed to strengthen intra‐cluster connectivity and weakens inter‐cluster connectivity from the perspective of gene dimension.

### 2.2 stDCL Accurately Identifies Spatial Heterogeneity from Spatial Transcriptomic Profiling of DLPFC

**Figure 2:**
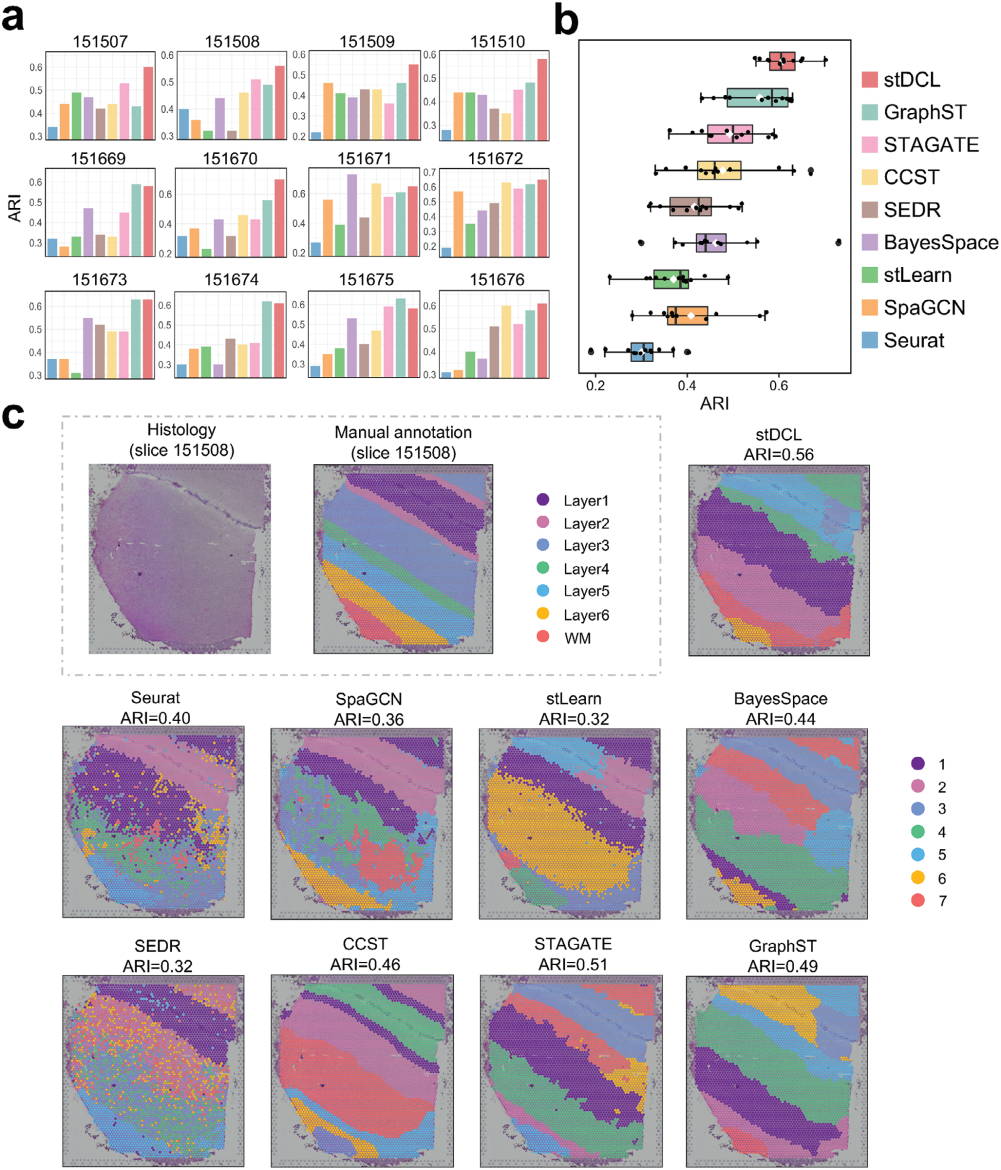
stDCL accurately identifies spatial heterogeneity from spatial transcriptomic profiling of DLPFC. a) Comparison of ARI values between stDCL and 8 spatial transcriptomics clustering methods on 12 DLPFC slices (*n* = 1 in each group). b) The box plot of clustering accuracy on all 12 DLPFC slice datasets in terms of ARI score for 9 methods (*n* = 12 in each group; center line, median; box limits, upper and lower quartiles; whiskers, 1.5× interquartile range). c) The manually annotated layer structure compared to spatial domains detected by Seurat, SpaGCN, stLearn, BayesSpace, SEDR, CCST, STAGATE, GraphST, and stDCL on slice 151508.

To evaluate the spatial clustering performance of stDCL, we used it on the human dorsolateral prefrontal cortex (DLPFC) 10x Visium dataset^[^ ^28^ ^]^ from brain tissue, which contains spatially resolved transcriptomic profiles of 12 DLPFC slices. The cortical layers (L1‐L6) and the white matter (WM) regions within each slice have been carefully identified by manual annotation, relying on morphological characteristics and genetic markers, as described by Maynard *et al.* ^[^ ^28^ ^]^ To ensure a comprehensive comparison, we conducted a comparative analysis of stDCL against eight state‐of‐the‐art methods (Seurat,^[^ ^29^ ^]^ SpaGCN,^[^ ^21^ ^]^ stLearn,^[^ ^19^ ^]^ BayesSpace,^[^ ^20^ ^]^ SEDR,^[^ ^30^ ^]^ CCST,^[^ ^23^ ^]^ STAGATE,^[^ ^22^ ^]^ and GraphST^[^ ^26^ ^]^) on the DLPFC dataset. **Figure** **2**a shows the results using the Adjusted Rand Index (ARI) metric to compare performance between stDCL and the other methods on the 12 tissue slices. We observe that stDCL achieved the highest ARI score on 8 of the 12 datasets. We depict the overall clustering performance of the different methods on the 12 tissue slices using boxplots, where the middle line and white diamond represent the median and mean ARI values, respectively. On all 12 slices, stDCL showed superior performance with the highest mean (ARI = 0.61) and median (ARI = 0.61) scores, exhibiting the least variance among the methods evaluated, as depicted in Figure 2b. GraphST also demonstrated remarkable spatial clustering performance, with a median score reaching 0.59. In contrast, the mean and median scores of the other methods were not above 0.50, with both BayesSpace and CCST displaying high variance, with significant performance fluctuations across the several tissue slices. For a more detailed assessment, we further utilized the normalized mutual information (NMI) and homogeneity score (HS) metrics to evaluate the clustering performance of stDCL (Figure S1, Supporting Information). Notably, the experimental results indicated that stDCL outperformed the other methods with both metrics.

We next take tissue slice 151508 as an example, which contains 4384 spots, both stDCL and STAGATE achieved superior clustering performance, and successfully identified the spatial domains that closely match the manually annotated tissue layers (Figure 2c). In contrast, methods such as Seurat and SEDR encountered difficulty in clearly defining layer boundaries, leading to numerous outliers that compromised the spatial structuring in the brain region. SpaGCN failed to separate the spots within layers L4‐L6 based on the hierarchical structure, whereas for stLearn and CCST, cluster most spots in layers L4‐L6 into a single expansive cluster. Overall, stDCL can effectively identify the cortical layer structures in the DLPFC dataset with stability and robustness across several slices. Figures S2– S4 (Supporting Information) detail the outcomes for the additional slices.

### 2.3 stDCL Provides Better Clustering Performance on Complex Spatial Domains and Tissue Structures

**Figure 3:**
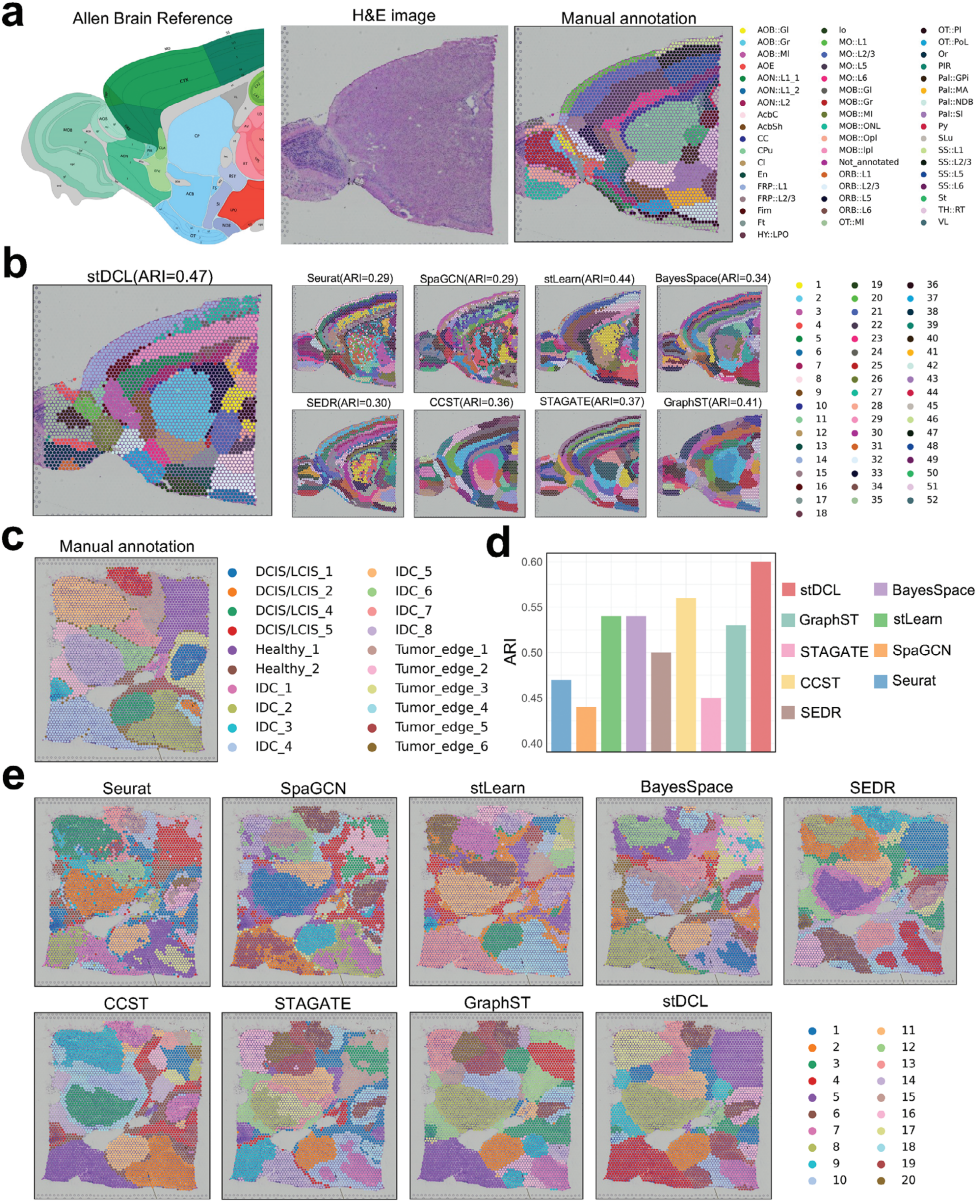
Spatial clustering performance on datasets with complex tissue structures.) The spatial domains of the mouse anterior brain dataset manually annotated by the Allen Mouse Brain Reference Atlas. b) The spatial domains detected by different methods on the mouse brain dataset. c) H&E images and manual annotation on the human breast cancer dataset. d) Comparisons of ARI values between stDCL and 8 spatial transcriptomics clustering methods on the human breast cancer dataset (*n* = 1 in each group). e) Spatial domains detected by different methods on the human breast cancer dataset.

In the next benchmark experiment, we applied stDCL to two 10x Visium datasets with more complex tissue structures (the mouse anterior brain dataset and the human breast cancer dataset), and compared it with other state‐of‐the‐art clustering methods. Long *et al.* ^[^ ^26^ ^]^ provided manual annotations for this mouse dataset in accordance with the Allen Mouse Brain Reference Atlas, identifying 52 clusters, as depicted in **Figure** **3**a. The experimental results revealed that stDCL outperformed the other methods in terms of clustering performance on this dataset (Figure 3b; Figure S5, Supporting Information). From the spatial clustering results, it becomes evident that only stDCL succeeded in accurately capturing the ‘CPu’ domain and fully identifying the “St” domain, while other methods produced aberrant tissue structures or disorganized spot distributions. Furthermore, the clusters identified by Seurat, SpaGCN, and SEDR showed spatial fragmentation, and the boundaries defined by stLearn and BayesSpace were indistinct. Of the remaining three methods, CCST and STAGATE introduced unnecessary layer structures, while GraphST formed discontinuous clusters. In summary, on spatial transcriptomics datasets with intricate organizational structures, stDCL demonstrated its capacity to effectively cluster spatial domains that align with biological annotations and identify several substructures.

For the human breast cancer dataset, the dataset was manually annotated with 20 regions by pathologists based on the H&E image and reported breast cancer marker genes (Figure 3c). stDCL still maintained the best clustering performance on this dataset. The ARI value of stDCL improved by 7% compared with other methods (Figure 3d), and it also outperformed other methods in the evaluation metrics of NMI and HS (Figure S6, Supporting Information). The spatial domains identified by stDCL were the closest to manual annotation (Figure 3e). Specially, for regions “IDC 5” and “Healthy 1,” only stDCL clearly captures most of the spots as a cluster, while other methods failed. Besides stDCL, CCST also had higher ARI value and better spatial clustering performance on the human breast cancer dataset, while Seurat, SpaGCN and STAGATE performed poorer clustering results on this dataset. In summary, on spatial transcriptomics datasets with complex organizational structures, scDCL was able to cluster spatial domains consistent with biological annotations and identify some substructures.

### 2.4 stDCL Efficiently Demarcates Tissue Structures in Spatial Transcriptomic Profiling Across Different Platforms

**Figure 4:**
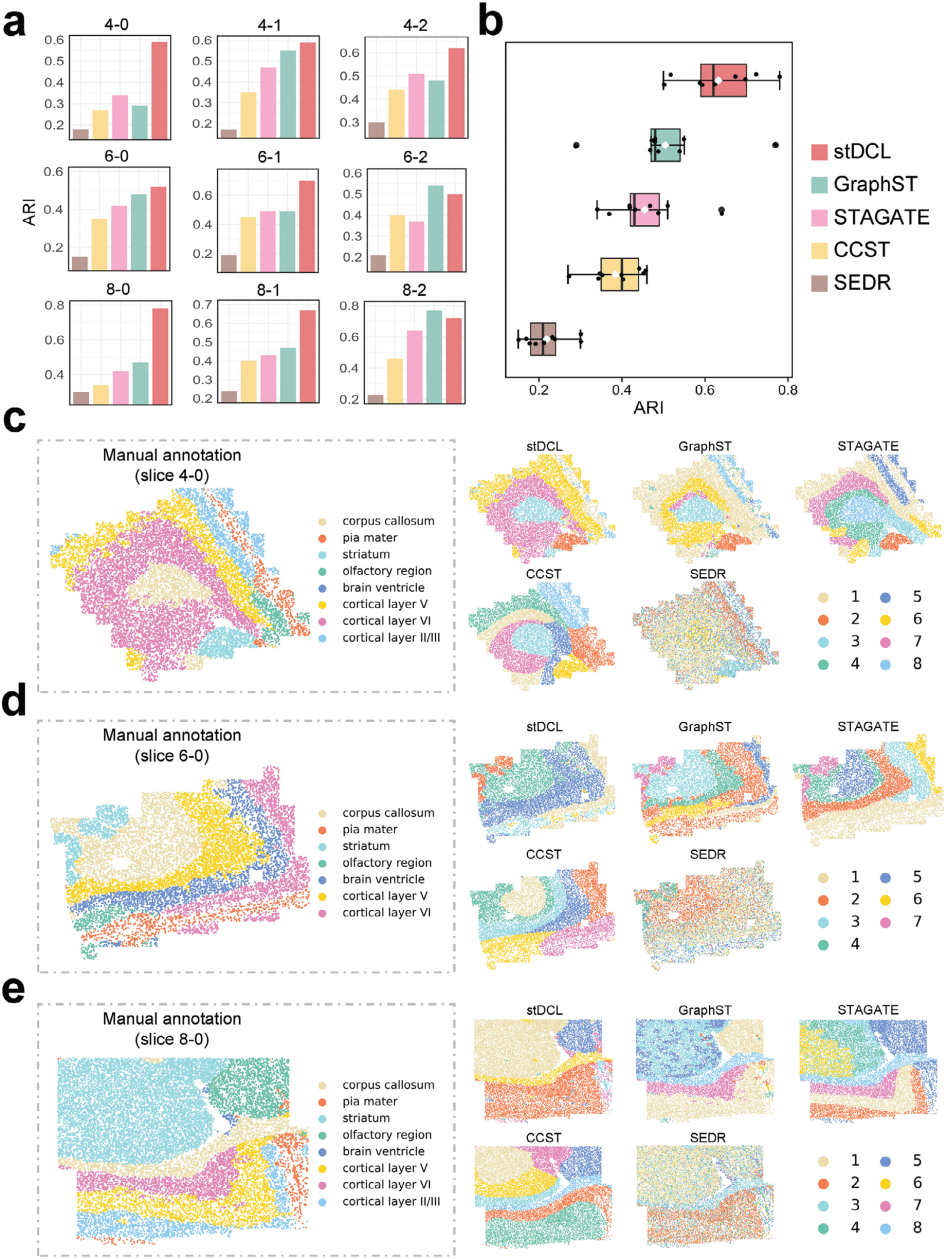
stDCL efficiently demarcates tissue structures in spatial transcriptomic profiling across different platforms. a) Comparisons of ARI values between stDCL and 4 spatial transcriptomics clustering methods on 9 slices from the MERFISH dataset (*n* = 1 in each group). b) The box plot of clustering accuracy on all 9 slice datasets in terms of ARI score (*n* = 9 in each group; center line, median; box limits, upper and lower quartiles; whiskers, 1.5× interquartile range). The manually annotated tissue structure compared to spatial domains detected by stDCL, GraphST, STAGATE, CCST and SEDR on c slice 4‐0, d slice 6‐0 and e slice 8‐0.

To test the spatial clustering performance of stDCL using various spatial transcriptomics technologies, we used it on different platforms to analyze the MERFISH dataset,^[^ ^31^ ^]^ with 9 tissue slices from the frontal cortex and striatum of mouse brain of diverse ages. From the 9 slices, slices 4‐0, 4‐1, 4‐2, 6‐0, 6‐1, and 6‐2 correspond to mouse brain at 4 weeks, and slices 8‐0, 8‐1, and 8‐2 are mouse brain at 90 weeks. Distinct tissue regions were manually delineated on each slice, indicating the corpus callosum, pia mater, striatum, olfactory region, brain ventricle, cortical layer V, cortical layer VI, and cortical layer II. We evaluated the performance of the algorithm on different platforms and further conducted a comparative analysis comparing stDCL with four well‐known spatial clustering methods, including GraphST, STAGATE, CCST, and SEDR. As depicted in **Figure** **4**a, stDCL demonstrated superior spatial clustering performance on all 9 tissue slices compared to other methods, with stDCL achieving the highest ARI on 7 of them. In terms of overall clustering performance, stDCL exhibited the highest mean score (ARI = 0.63) and median (ARI = 0.62) of all methods across all 9 slices (Figure 4b). Notably, stDCL attained an ARI of 0.78 on slice 8‐0, significantly outperforming the other methods. The mean and median scores of the other methods were below 0.55, with SEDR proving ineffective for spatial clustering on the MERFISH dataset. Additionally, we employed NMI and HS metrics to provide a comprehensive assessment of the performance of stDCL on the MERFISH dataset (Figure S7, Supporting Information). The experimental findings indicated that stDCL surpasses the other methods measured by both metrics, and notably, stDCL achieved the highest HS scores on all slices.

In the young mouse brain slices 4‐0 and 6‐0, and in an old brain slice 8‐0, stDCL accurately identified the spatial domains, aligning closely with the manually annotated tissue structures (Figure 4c–e), distinguishing regions like the corpus callosum, pia mater, and striatum in both ages, which was challenging for the other methods. Specifically, the pia mater identified by GraphST tended to be conflated with other tissue structures, and the clarity of the striatum identification in old slice 8‐0 was markedly compromised compared to stDCL. STAGATE and CCST, failed to identify the pia mater, and the corpus callosum identified in young brain slice 6‐0 and striatum in old brain slice 8‐0 were incomplete. The spatial domains found by SEDR are relatively disordered and the spatial clustering performance poor. Moreover, Figures S8–S10 (Supporting Information) provide results for all other slices for an exhaustive evaluation. We aslo applied stDCL to the slices from Adult mouse central nervous system, obtained using the STARMap Plus technique.^[^ ^32^ ^]^ Distinct tissue regions were manually delineated on three slice, indicating the CB 1, CB 2, CTX 1, CTX 2, DG, and so on. As depicted in Figure S11a (Supporting Information), stDCL demonstrated superior spatial clustering performance on all 3 tissue slices compared to other methods. In mouse central nervous system slices, stDCL accurately identified spatial domains that were tightly aligned with manually annotated tissue structures (Figure S11b, Supporting Information). Notably, stDCL is able to successfully detect HY regions, whereas other methods either failed to detect or incorporated HY regions into other regions. For the other methods, GraphST is also able to identify most of the spatial domains, while CCST and SEDR detected regions that are more ambiguous. In conclusion, stDCL consistently exhibits effective performance in spatial clustering on several datasets utilizing various spatial transcriptomics technologies, and maintaining robust performance with different age of mouse brain.

We also compared the runtime of stDCL with other clustering algorithms. For the test datasets, we adopted 19 datasets with 1200–30 000 cells (Figure S12, Supporting Information). As shown in Figure S12 (Supporting Information), stDCL, GraphST, and SEDR demonstrate comparable execution times, while CCST exhibits the longest runtime among the methods. Notably, CCST's runtime escalates sharply once the cell count surpasses 5000, indicating a steep increase in computational demand at higher cell densities. For STAGATE, the growth rate in running time is particularly pronounced between 10 000 and 20 000 cells, highlighting its sensitivity to increased cell numbers. Although stDCL's runtime is generally longer than that of GraphST, this is attributed to the added computational complexity introduced by its dual graph contrastive learning mechanism, which enhances model performance at the cost of increased processing time. In addition, we compared the memory usage of these methods at different numbers of cells (Figure S13, Supporting Information). In Figure S13 (Supporting Information), we can observe that stDCL and GraphST maintain relatively consistent memory usage, even as the number of cells scales up, suggesting their efficiency in managing computational resources. However, CCST has significantly higher memory requirements, especially when the number of cells exceeds 5000, suggesting that its algorithmic structure may need to allocate a large amount of memory for larger datasets. SEDR maintains a lower memory usage. As the number of cells increases from 20 000 to 25 000, STAGATE's memory usage also increases significantly, reflecting the method's growing demand on resources for larger data sizes. In summary, despite incorporating dual graph contrastive learning, stDCL maintains runtime and memory usage within a manageable range.

### 2.5 The Latent Embedding Characterization of stDCL Aggregates Interpretable Spatial Expression Information

**Figure 5:**
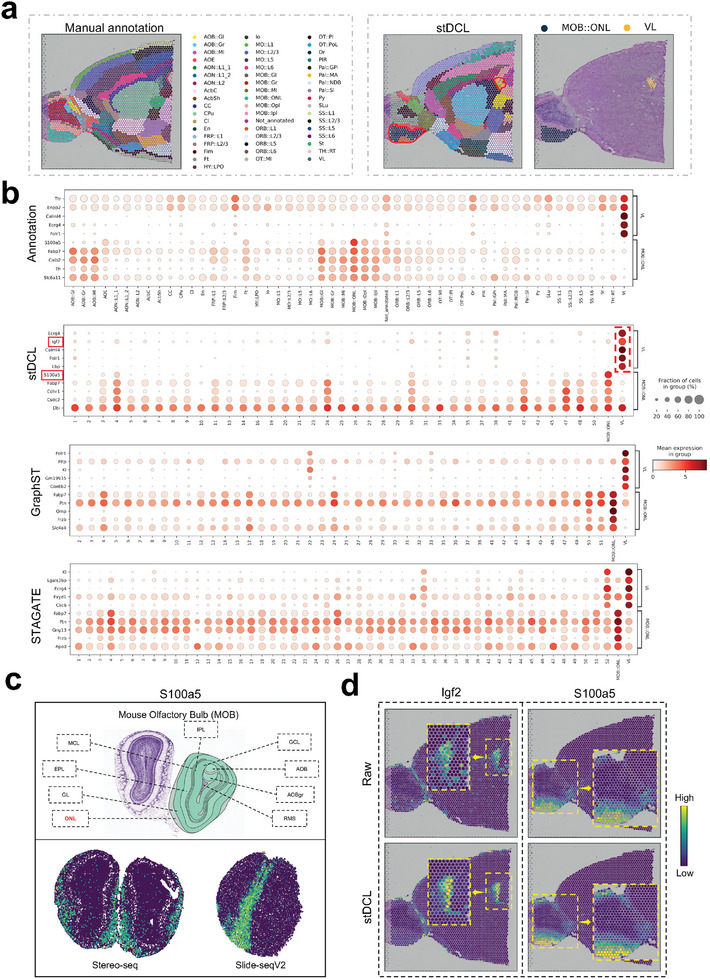
The latent embedding characterization of stDCL aggregates interpretable spatial expression information. a) The spatial domains of the mouse anterior brain dataset manually annotated by the Allen Mouse Brain Reference Atlas (left). b) The dotplot of the top 5 highly expressed genes in MOB::ONL and VL in manual annotation, stDCL, GraphST and STAGATE. c) The visualization of expression of *S100a5* on mouse olfactory bulb datasets, profiled by Stereo‐seq and Slide‐seqV2, respectively. d) Visualization of the spatial expression of genes *Igf2* and *S100a5* denoised by stDCL.

To investigate whether stDCL can capture critical spatial transcriptomics information, we conducted genome interpretability analysis on the latent embedding representation of stDCL using the mouse forebrain 10x Visium dataset. Initially, we identified two regions based on the manual annotation, namely MOB::ONL and VL (**Figure** **5**a). We then utilized an interpretable gene selection method^[^ ^33^ ^]^ informed by the standard deviation ranking of GNN weight matrices to select the top 1000 most highly expressed genes from the latent embedding representations of stDCL, GraphST, and STAGATE. Subsequently, we visualized the top 5 most highly expressed genes in regions MOB::ONL and VL using the spatial clustering methods and compared with the highly expressed genes obtained by manual annotation (Figure 5b). It can be observed that stDCL correctly identified the highly expressed genes in the VL region without mistaking genes from other spatial domains. Furthermore, stDCL detected highly expressed genes, such as *Igf2*, which were not identified by the other methods or even the manual annotation. For the olfactory bulb (MOB) olfactory nerve layer (ONL) MOB::ONL region, both stDCL and manual annotation found the highly expressed gene *S100a5*, while the other methods failed. To verify the significance of *S100a5* expression in the MOB::ONL region, we visualized the expression of *S100a5* in the mouse olfactory bulb datasets profiled by Stereo‐seq^[^ ^34^ ^]^ and Slide‐seqV2,^[^ ^35^ ^]^ respectively (Figure 5c). As illustrated in Figure 5c, the *S100a5* gene is clearly highly expressed in the olfactory nerve layer. Recent studies have also demonstrated that *S100a5* is mainly expressed in the olfactory bulb and olfactory sensory neurons (OSNs), with expression significantly upregulated in response to olfactory stimulation.^[^ ^36^, ^37^ ^]^

To further evaluate the ability of stDCL to aggregate and characterize the spatial expression information of the data, we computed the 15 nearest neighbors for each spot based on the Euclidean distance in the latent embedding representation of stDCL and then averaged the gene expression data for these neighbors to yield a denoised gene expression matrix. As depicted in Figure 5d, compared to the raw gene expression data, the expression levels of the interpretable genes *Igf2* and *S100a5*, identified through the latent embedding representation, were elevated in their respective regions, and their enrichment levels were higher. Overall, these experimental results demonstrate that the latent embedding characterization of stDCL can uncover important interpretable genes and aggregate their spatial expression information, offering insights into the molecular architecture and function of tissue.

### 2.6 stDCL Efficiently Recovers Gene Expression to Characterize Spatial Hierarchy and Differential Gene Expression in the Mouse Visual Cortex

**Figure 6:**
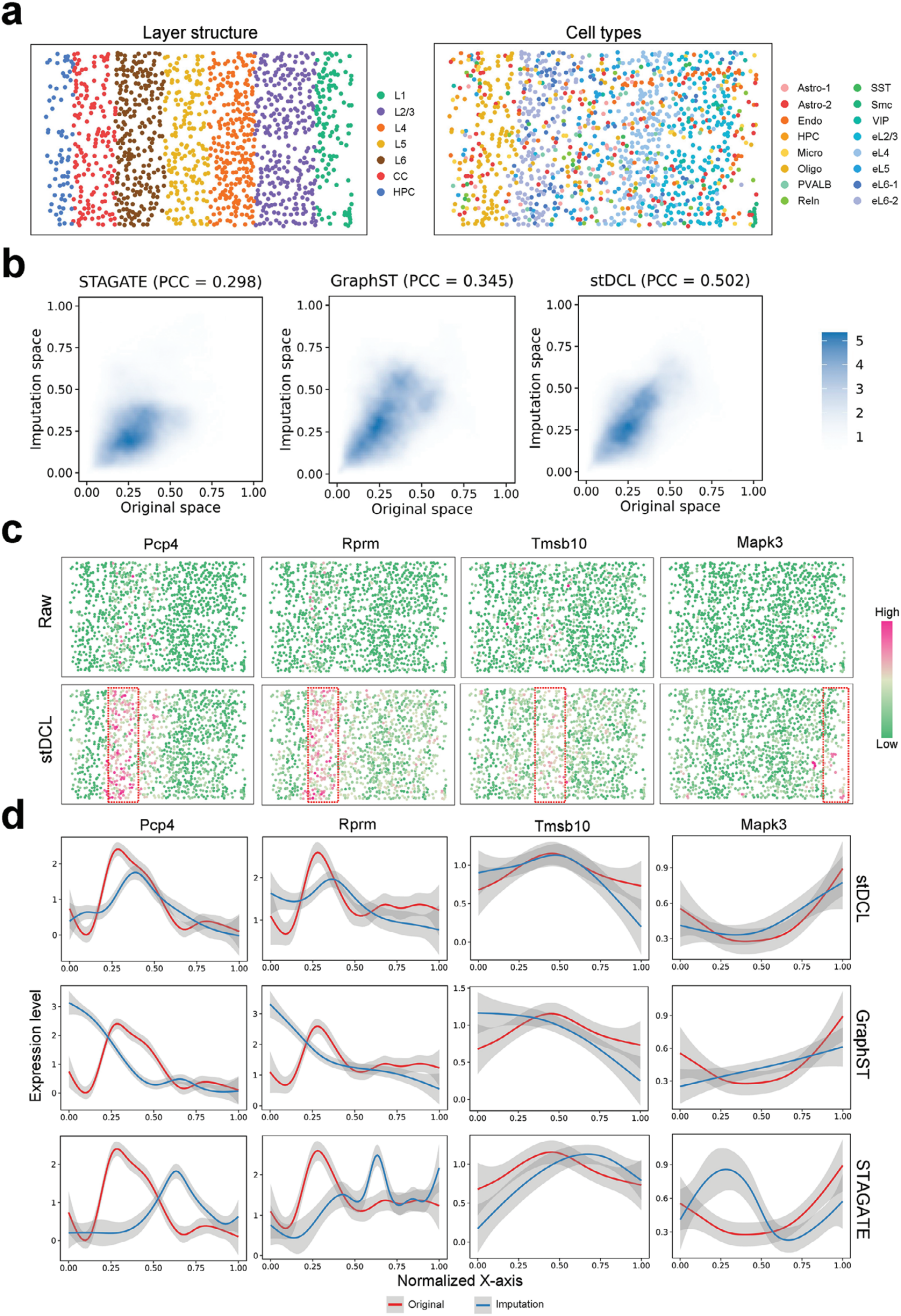
stDCL can efficiently recover gene expression to characterize spatial hierarchy and differential gene expression in the mouse visual cortex. a) The layer structure and cell types of the tissue slices of the mouse primary visual cortex (V1) dataset. b) The density plot of the correlation of pairwise distances between spots in the original space and the imputation space generated by STAGATE, GraphST and stDCL. c) The spatial expression of layer‐specific genes. d) The expression trend of differential genes in each layer along the *X*‐axis in the original space and the imputation spaces generated by STAGATE, GraphST and stDCL, respectively.

To investigate the reconstruction performance of stDCL in spatial hierarchy structure and denoising and enhancement of differential gene expression, we used mouse primary visual cortex (V1) profiled by STARmap.^[^ ^38^ ^]^ The dataset comprises 1,207 cells and 1,020 genes, and the layer structure and cell types of the tissue slices are depicted in **Figure** **6**a. We first applied STAGATE, GraphST, and stDCL to the STARmap dataset and compared their clustering performance (Figure S14, Supporting Information). The results demonstrated that stDCL surpassed the other methods. To evaluate the performance on reconstructing the hierarchical structure through the original spatial information, we first projected the imputation matrix generated by the three methods onto two dimensions as the imputation space using the scanpy package. We proceeded to compare the correlation of pairwise distances in the imputation space generated by stDCL, GraphST, and STAGATE with the original space, as depicted in Figure 6b. We see that the imputation space of stDCL exhibited a significantly higher correlation with the original space compared to the other two methods, with a Pearson correlation coefficient (PCC) of 0.502, while STAGATE and GraphST achieved PCC values of 0.298 and 0.345, respectively. We also used other correlation metrics for systematic comparison, and stDCL is the best in all four metrics (Figure S15, Supporting Information). In addition, the imputation space of stDCL preserved the relative position between the L1‐L6 layers, that is, the distance to the L1 layer gradually increased from the inside to the outside, whereas the hierarchies reconstructed by STAGATE and GraphST did not show this tendency (Figure S16, Supporting Information).

We also assessed the performance of stDCL imputation on denoising and enhancing differential gene expression results. Specifically, we focused on four layer‐specific differential genes, *Pcp*4 (L6), *Rprm* (L6), *Tmsb*10 (L5) and *Mapk*3 (L1), and compared their spatial expression levels in both the raw and imputed expression matrices (Figure 6c; Figure S17, Supporting Information). These genes were specifically expressed at the corresponding spatial positions of the imputation matrix, whereas their raw spatial expression was inconspicuous and dispersed. We further investigated the expression trends of these layer‐specific genes along the *X*‐axis in the imputation space and the original space. As illustrated in Figure 6d, the imputation space of stDCL effectively preserved most of the expression patterns of genes along the layer axis, closely resembling the patterns observed in the original space. For other methods, GraphST retained only a part of the gene expression trend, while STAGATE displayed a broader trend. In particular, all three methods substantially preserved the expression pattern of the gene *Tmsb*10. Overall, imputation of stDCL not only successfully reconstructs the hierarchical structure of the spatial transcriptomics data, but also clarifies and strengthens the identification of layer‐specific differential genes to unravel spatial intricacies in the mouse visual cortex.

### 2.7 Evaluation of Hyperparameter Selection and Ablation Analysis

We evaluated the effect of the number of selected genes, the number of neighbors and their metric distance used to construct the neighborhood graph, the loss weights, the network framework and other parameters on the clustering performance of stDCL on 12 DLPFC slice datasets. In addition, we investigated the effectiveness of each component of stDCL on the same dataset.

**Figure 7:**
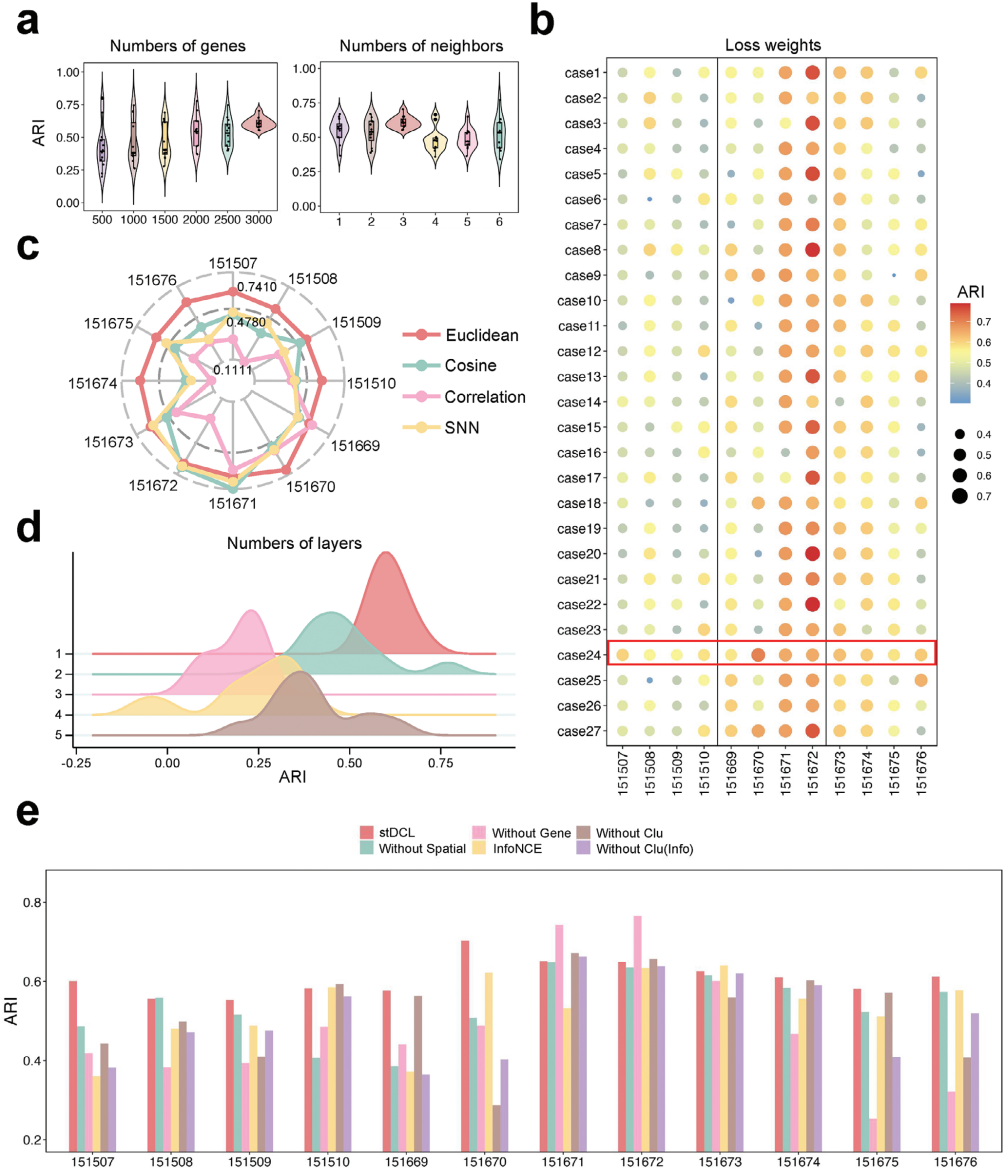
Evaluation of hyperparameter selection and ablation analysis on 12 DLPFC slices. a) The clustering performance was evaluated using ARI metric for different numbers of genes and numbers of neighbors (*n* = 12 in each group; center line, median; box limits, upper and lower quartiles; whiskers, 1.5× interquartile range). b) The ARI bubble plot under different loss weight assignments. c) The radar plot of clustering performance for different ways and distance metrics of constructing the neighborhood graph. d) The ridgeline plot of clustering performance for different numbers of hidden layers in the graph autocoder (*n* = 12 in each group). e) The ablation study for the neighborhood graph and dual graph contrastive learning (*n* = 1 in each group).

The clustering performance of stDCL was evaluated using varying numbers of selected genes and different numbers of neighbors, as depicted in **Figure** **7**a and Figure S18a (Supporting Information). The results revealed that stDCL achieved optimal clustering when utilizing 3000 genes as input and constructing the neighborhood graph with three neighbors. Subsequently, stDCL was trained by three loss functions simultaneously, namely reconstruction loss, space‐aware contrastive loss, and cluster‐level feature contrastive loss. To ensure a harmonious balance of the overall loss function, we assigned weights to three parameters (γ_1_, γ_2_, and γ_3_) and explored various combinations of weights. Specifically, in accordance with our previous experiences, we selected γ_1_, γ_2_, and γ_3_ in the range [1.0, 5.0, 10.0], [0.2, 0.5, 0.8], or [0.2, 0.5, 0.8]. On this basis, we enumerated weights to obtain 27 distinct loss weight assignments and compared the clustering performance in each case (Figure 7b). Notably, among the 27 configurations, composition 24 yielded the highest average ARI value, where γ_1_, γ_2_, and γ_3_ were set to [10, 0.5, 0.8], respectively. We also employed NMI and HS metrics for further comparisons (Figure S19, Supporting Information). We also systematically analyze the parameters *alpha*_1_ and *alpha*_2_ of the reconstruction loss and give the best settings as [1.0, 0.5] (Figure S20, Supporting Information). To construct the neighborhood graph, we tested the clustering performance using different methods and distance metrics. As shown in Figure 7c and Figure S18b (Supporting Information), stDCL achieved optimal clustering performance when constructing a KNN graph using the Euclidean distance metric. Regarding the network architecture, we evaluated the clustering performance of stDCL with varying numbers of hidden layers, as depicted in Figure 7d and Figure S18c (Supporting Information). Experimental results indicated that stDCL was most effective in clustering with a single hidden layer. In addition, we also analyzed the number of nodes in the hidden layer, as illustrated in Figure S21 (Supporting Information).

We conducted an ablation analysis on stDCL to assess the effectiveness of the neighborhood graph and dual graph contrastive learning mechanisms. Specifically, we compared the spatial clustering performance of stDCL with its partially ablated counterpart using the ARI metric. In the neighborhood graph module construction, we removed two components: spatial location (“Without Spatial”) and the gene expression matrix (“Without Gene”). In the contrastive learning module, we evaluated three ablation cases: (1) replacing the space‐aware contrastive loss function with the InfoNCE loss function (“InfoNCE”); (2) removing the cluster‐level feature contrastive loss function (“Without Clu”): (3) using only the InfoNCE loss function (“Without Clu(Info)”). As shown in Figure 7e and Figure S22 (Supporting Information), the spatial clustering performance of stDCL outperformed the other arrangements. In addition, the clustering performance of stDCL exhibited greater stability, whereas the other arrangements displayed some degree of fluctuation. In particular, the spatial clustering performance of stDCL after removing gene expression information during the construction of the neighborhood graph was significantly higher than that of the other arrangements on slices 151671 and 151672. This implies that spatial information plays a predominant role in the correlation of spots on these two slices, and the addition of gene expression information has an adverse impact on clustering performance. In summary, these experimental results highlight the effectiveness of both the neighborhood graph construction and the dual graph contrastive learning components in stDCL. To provide a more comprehensive analysis, we investigated how dual graph contrastive learning with stDCL contributes to the performance of various tasks, including spatial heterogeneity dissection, spatial hierarchy characterization and gene expression recovery. We evaluated three ablation cases: (1) using only the space‐aware contrastive learning (stDCL(Spa)); (2) using only the cluster‐level feature contrastive learning (stDCL(Clu)): (3) using only the InfoNCE loss function (stDCL(Info)). We study the performance of these cases on various tasks in more detail and provided the analysis and experimental results in Note S8 (Supporting Information).

### 2.8 stDCL Reveals Molecular and Spatial Heterogeneity in Mouse Brain at Single‐Cell Resolution

**Figure 8:**
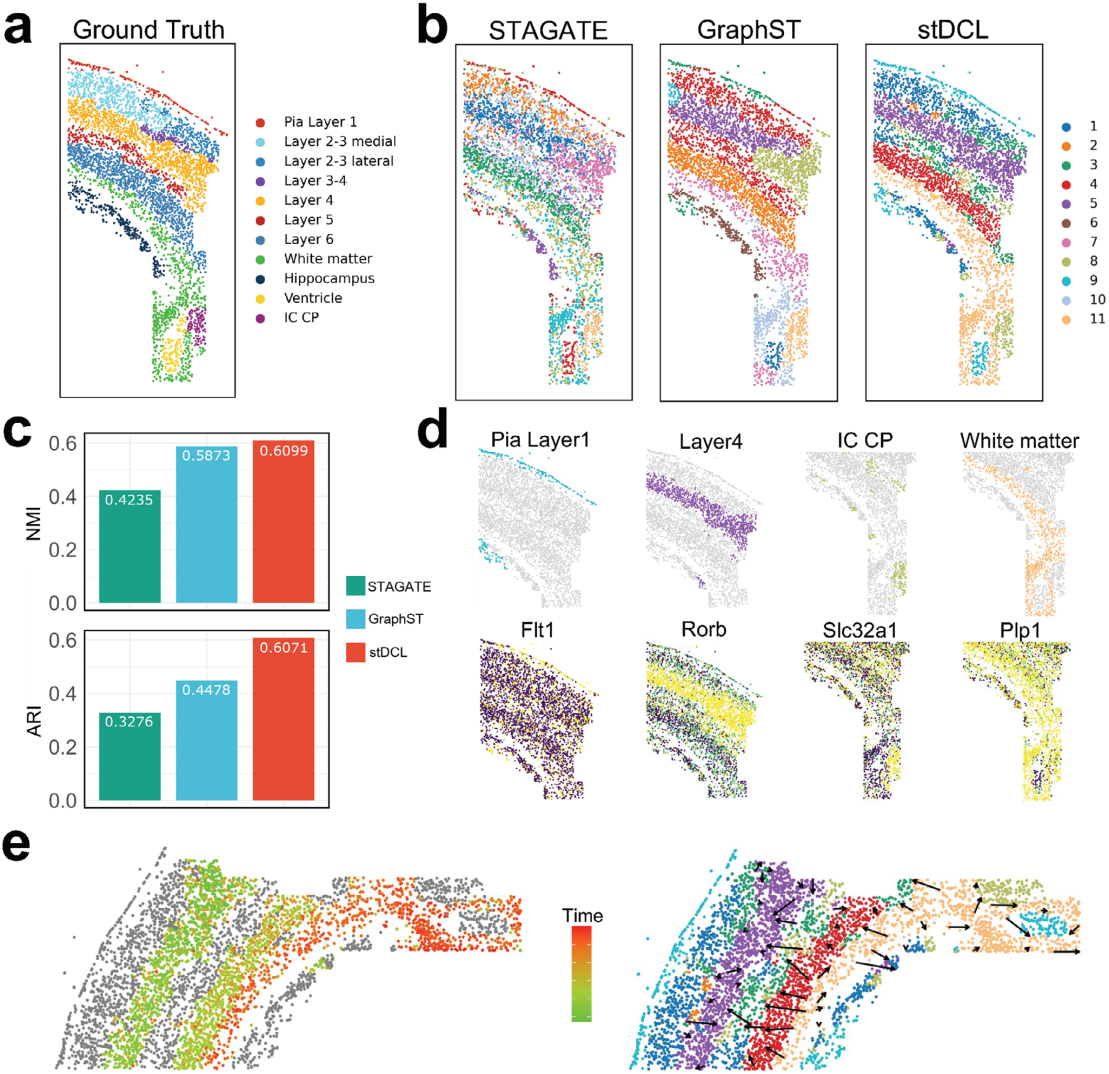
stDCL reveals molecular and spatial heterogeneity in mouse brain at single‐cell resolution. a) The ground truth of regional annotation for the osmFISH dataset. b) The spatial domains identified by STAGATE, GraphST and stDCL. c) The clustering performance comparison of the three methods. d) The visualization of domains identified by stDCL and the corresponding marker genes. e) The visualization of the trajectory inferred by stDCL.

To evaluate whether stDCL can explore molecular and spatial heterogeneity, we applied stDCL to analyze spatial transcriptomics data from the mouse brain at single‐cell resolution. We collected a spatial dataset obtained from the mouse somatosensory cortex acquired with osmFISH,^[^ ^39^ ^]^ which contains 4839 cells and 33 genes. The real annotations of this dataset, as illustrated in **Figure** **8**a, exhibit its origin at the Pia Layer 1 and encompass 11 cell‐wise regions. We compared the spatial clustering performance of STAGATE, GraphST and stDCL on this dataset using NMI and ARI metrics, and then visualized the clustering results (Figure 8b,c, Supporting Information). The experimental results indicate that stDCL significantly outperformed other methods, and the identified spatial domains are closer to the real annotation. To elaborate, in comparison to the other two methods, stDCL could distinguish Layer 4 and White matter more effectively, rendering the layer boundaries more distinctly defined. Furthermore, we validated that stDCL revealed heterogeneity in these spatial layers through the expression of marker genes^[^ ^39^ ^]^ native to osmFISH data (Figure 8d). For instance, *Rorb*, a marker gene for Layer 4 pyramidal neurons, exhibited the expected high expression within Layer 4 identified by stDCL. *Plp1*, a marker gene for oligodendrocytes, displayed elevated expression within the white matter, where various types of oligodendrocytes are known to reside. In addition, *Flt1*, a marker gene for endothelial cells in Pia Layer 1, and *Slc32a1*, a marker gene for interneurons in IC CP, were also found to be expressed as anticipated within their respective spatial locations. These findings collectively emphasize the capacity of stDCL to elucidate the molecular and spatial heterogeneity, further revealing spatial gene expression patterns. In addition, we also conducted trajectory inference on the relatively well‐defined domains identified by stDCL, such as Layer 4, Layer 6, and white matter (Figure 8e). The inferred trajectory projects from White matter to Layer 4, aligning with the established pattern of cortical development from the inside out,^[^ ^40^, ^41^ ^]^ where new neurons migrate vertically along the radial glial fibers to the marginal area of the cortical periphery to form a new cortex on top of the existing layers.

**Figure 9:**
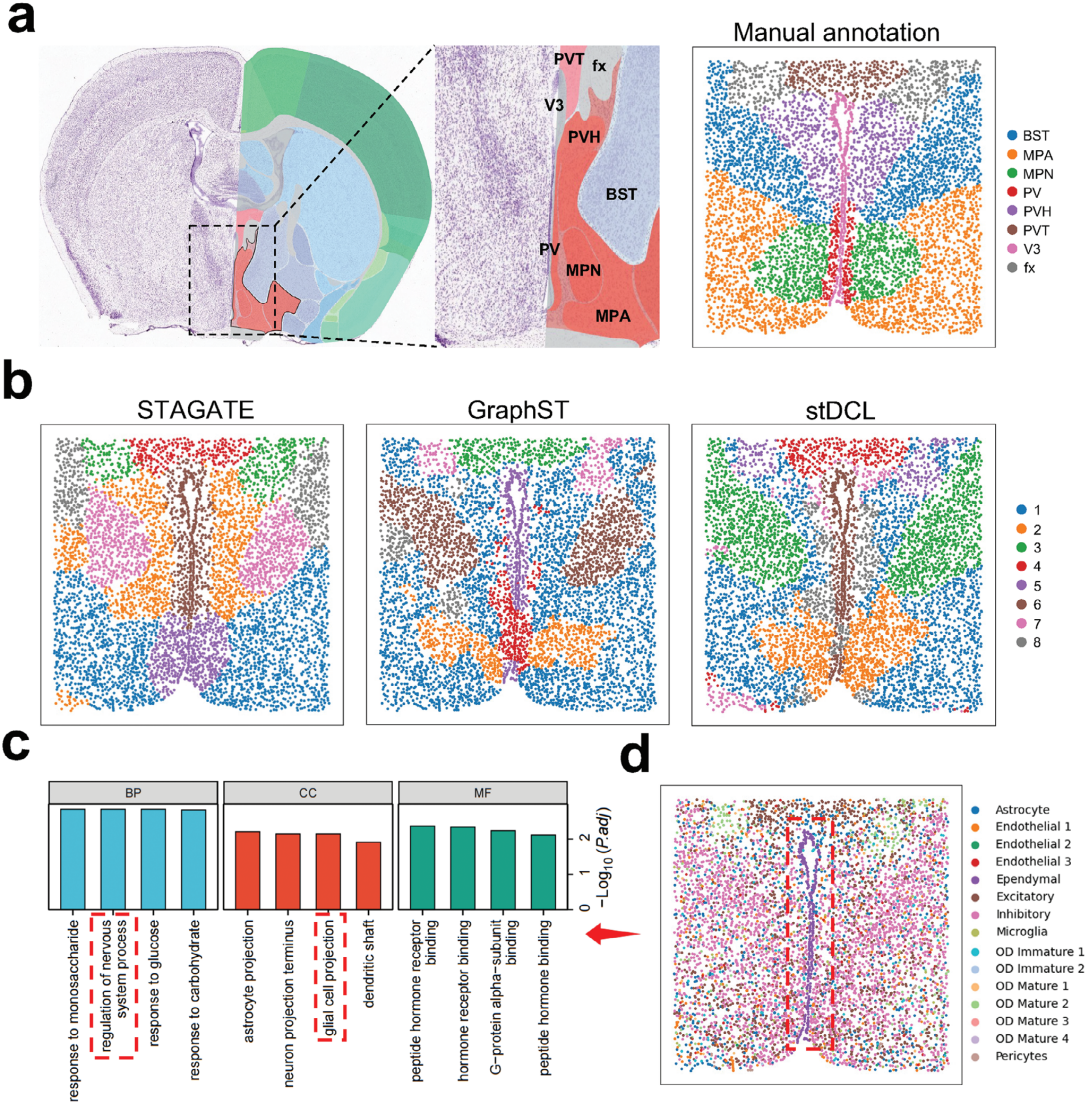
stDCL reveals molecular and spatial heterogeneity in mouse brain at single‐cell resolution. a) The manual annotation of mouse hypothalamic regions with reference to the Allen Mouse Brain Atlas. b) The identified spatial domains are shown for STAGATE, GraphST and stDCL. c) The GO enrichment analysis (two‐sided Wilcoxon test and adopt BH to adjust *p*‐values for multiple comparisons). d) The true cell annotation of mouse hypothalamic MERFISH dataset.

Subsequently, we conducted an analysis of mouse hypothalamic preoptic area tissue slices (Bregma‐0.14) obtained using the MERFISH Spatial Profiling Technology.^[^ ^42^ ^]^ This dataset contains 5926 cells and 155 genes, annotated across eight structural regions: bed nuclei of the strata terminalis (BST), medial preoptic area (MPA), medial preoptic nucleus (MPN), periventricular hypothalamic nucleus (PV), paraventricular hypothalamic nucleus (PVH), and paraventricular nucleus of the thalamus (PVT), third ventricle (V3), and columns of the fornix (fx) (**Figure** **9**a). We examined the spatial domain clustering results of STAGATE, GraphST, and stDCL (Figure 9b). It can be observed that stDCL surpassed the other methods in its ability to detect the majority of spatial domains, exhibiting a closer resemblance to the histological annotations. Specifically, stDCL was adept at identifying and delineating the BST region, achieving smoother boundaries than those produced by alternative approaches. In addition, stDCL was able to successfully identify the V3 region, a task that STAGATE failed to perform in the lower part of the V3 region, and GraphST incorrectly merged some V3 cells with other regions. Then, we performed enrichment analysis on the top 20 differentially expressed genes detected in the V3 region identified by stDCL (Figure 9c) and detected marker genes for this region (Figure S26, Supporting Information). These genes are mainly enriched in response to glucose and monosaccharide; the reason being there are basically ependymal cells in the V3 region (Figure 9d), suggesting their potential involvement in glucose and monosaccharide transport.^[^ ^43^, ^44^ ^]^ Moreover, ependymal cells, as a type of glial cell found in the central nervous system, are an indispensable component of the central nervous system.^[^ ^45^ ^]^ Therefore, the V3 region is also enriched for Gene Ontology (GO) terms associated with glial cells and the nervous system, such as glial cell projections and regulation of nervous system processes. In summary, stDCL demonstrated an exceptional capacity to accurately identify and characterize the complex molecular and spatial heterogeneity embedding within the mouse brain at the single‐cell resolution.

### 2.9 stDCL Unravels Developmental Patterns in the Mouse Embryo Brain

**Figure 10:**
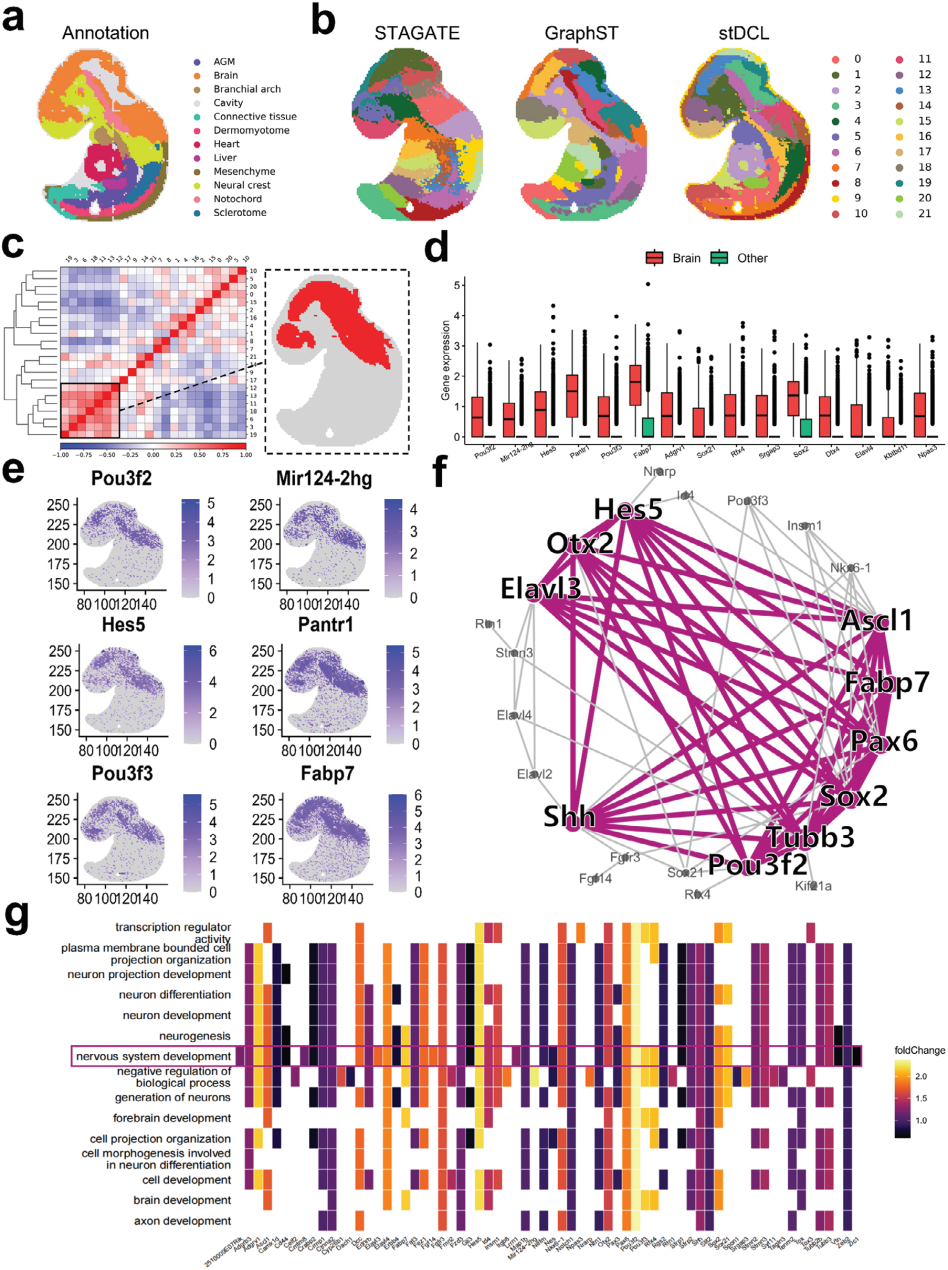
stDCL unravels developmental patterns in the mouse embryo brain. a) The ground truth of regional annotation for the E1S1 section of E9.5 embryos. b) The spatial domains identified by STAGATE, GraphST and stDCL. c) The correlation matrix between clusters identified by stDCL. A group of highly correlated clusters concentrated in the brain region is marked by a black box. d) The violin plot of expression levels of the top 15 up‐regulated genes. e) The visualization of expression levels of the top up‐regulated genes in the brain region identified by stDCL. f) The PPI network of up‐regulated differential genes. The purple marked part is the highest scoring gene module in the network calculated by MCODE. g) The heat map of GO enrichment analysis.

To investigate developmental patterns in the mouse embryonic brain, we applied stDCL to analyze multiple mouse embryo Stereo‐seq datasets^[^ ^34^ ^]^ acquired at the E9.5 and E10.5 time points. We first performed clustering analysis on the E1S1 slices of E9.5 embryos using STAGATE, GraphST, and stDCL, followed by a comparison with the ground truth annotations (**Figure** **10**a,b). The reason for setting the number of clusters higher than the number of annotated tissue regions was to obtain higher resolution identification of tissue regions. As depicted in Figure 10b, the clustering results of stDCL exhibited a closer alignment with the annotations, enabling the clear detection of specific regions such as heart, liver, and dermatomyositis. In addition, we analyzed the correlation between the clusters identified by stDCL. Notably, stDCL uncovered a group of highly correlated clusters concentrated in the brain region (Figure 10c). To explore developmental patterns in the mouse embryonic brain, we integrated this group of clusters as a significant brain region detected by stDCL and investigated its distinctions from other regions. We first calculated the differentially expressed genes within this brain region and visualized the expression levels of the top 15 up‐regulated genes (Figure 10d,e; Figure S27, Supporting Information). These genes are clearly highly expressed in the brain region compared to other regions. We further constructed a Protein–Protein Interaction (PPI) network for the up‐regulated differentially expressed genes via STRING^[^ ^46^ ^]^ and extracted the top‐scoring gene modules from the network using Molecular Complex Detection (MCODE)^[^ ^47^ ^]^ (Figure 10f). Additionally, we conducted Gene Ontology (GO) enrichment analysis, revealing that the highest‐scoring gene modules were associated with neurodevelopment (Figure 10g). In the early stages of mouse brain development, many neurons are generated, thus nervous system development plays an important role at this time.^[^ ^48^ ^]^ Moreover, several pathways related to brain development, including neuronal development, neuronal differentiation, and forebrain development, were also enriched. These findings indicate that stDCL reveals the developmental patterns inherent in the mouse embryo.

### 2.10 stDCL Uncovers Biological Connections Across Multiple Mouse Embryo Slices

**Figure 11:**
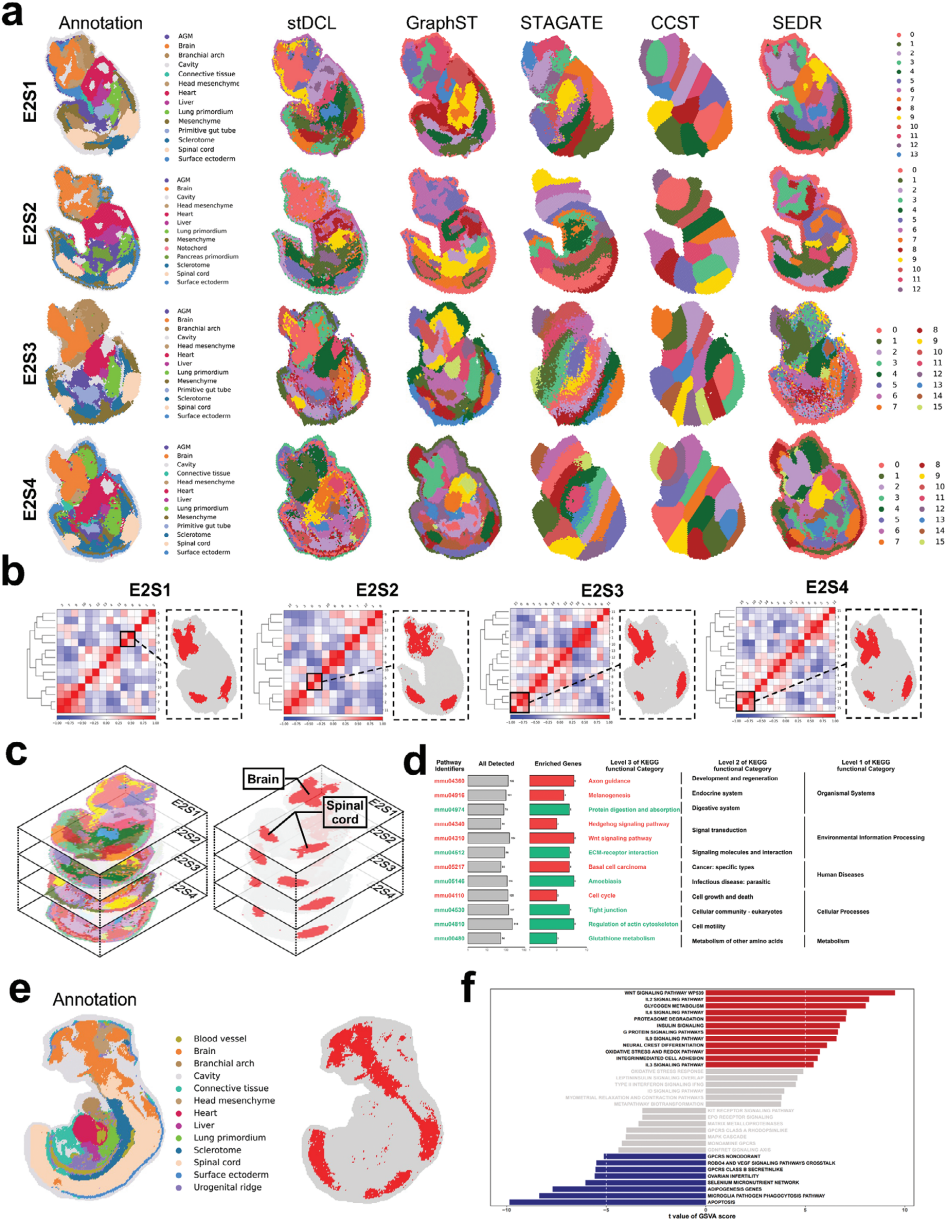
stDCL uncovers biological connections across multiple mouse embryo slices. a The ground truth of regional annotation for the E1S1, E2S1, E2S2, E2S3, and E2S4 section of E9.5 embryos. The spatial domains identified by stDCL, GraphST, STAGATE, CCST and SEDR. b The correlation matrix between clusters identified by stDCL. Marked by the black box is a group of highly correlated clusters concentrated in the brain region. c The spatial domains identified by stDCL on four sections of E2S1, E2S2, E2S3, and E2S4 in E9.5 embryos (left). The regions of correlation between the brain and spinal cord identified by stDCL on all four sections (right). d The KEGG pathway enrichment analysis of the selected region on the E2S1 section. e The ground truth of regional annotation for the E1S1 section of E10.5 embryos (left). The regions of correlation between the brain and spinal cord identified by stDCL on the E1S1 section (right). f The GSVA enrichment pathway diagram of the selected region.

We performed an extensive clustering analysis on four distinct embryonic slices, denoted as E2S1, E2S2, E2S3, and E2S4, from E9.5 embryos (**Figure** **11**a; Figure S28, Supporting Information). The experimental results show that the clustering performance of stDCL was better than other clustering methods overall and can correctly identify most regions. On this basis, we examined the correlation between the clusters identified by stDCL on these embryo sections (Figure 11b). Remarkably, stDCL revealed a consistent correlation between the regions identified within the brain and those present within the spinal cord on all four slices (Figure 11c). To investigate the underlying biological significance, we aggregated these regions for KEGG pathway analysis of differentially expressed genes within the integrated regions (Figure 11d; Figure S29, Supporting Information). It can be observed that up‐regulated differentially expressed genes are enriched for axon guidance and the Wnt signaling pathway in all four sections. This observation highlights their pivotal roles in axon guidance and Wnt signaling in the complex process of neuronal development, particularly in forming commissural circuits connecting the brain and spinal cord within the CNS.^[^ ^49^ ^]^ The axon guidance mechanism is key to accomplishing this task.^[^ ^50^, ^51^ ^]^ In addition, the Wnt signaling pathway is also essential for the formation of neuronal circuits.^[^ ^52^ ^]^ To elucidate further, the Wnt signaling controls neuronal polarity, promotes axon growth, and regulates axon navigation to the final synaptic target.^[^ ^53^, ^54^ ^]^

To provide a more comprehensive analysis, we extended our investigation by conducting analogous clustering and correlation analyses on the E1S1 section of E10.5 embryos, which has a denser spinal cord region distribution (Figure 11e; Figure S30, Supporting Information). We also found the consistent correlation between brain and spinal cord regions on this slice, and the area of the correlated region was larger. We performed gene set variation analysis (GSVA) on the integrated regions and found that the Wnt signaling pathway was predominantly enriched (Figure 11f). This demonstrates that embryos at this stage, which have a denser spinal cord region, are important for the formation of commissural circuits connecting the brain and spinal cord within the CNS. In particular, we also enriched for various Interleukin (IL) signaling pathways, including IL‐2, IL‐3, IL‐6, and IL‐9. The IL signaling pathways highlight the importance of immune‐neural interactions, especially in response to injury or inflammation. This is particularly relevant for areas such as the hypothalamus and spinal cord, which are involved in both neuroendocrine regulation and immune responses. For example, in the spinal cord, IL‐6 is involved in the regulation of chronic pain, especially neuropathic pain. It can promote inflammatory responses in the spinal cord by activating microglia and astrocytes, thereby affecting pain transmission.^[^ ^55^ ^]^ Collectively, these findings revealed that stDCL uncovers the biological connections between the brain and spinal cord in the mouse embryo.

### 2.11 stDCL Illustrates Underlying Regulatory Mechanisms in Alzheimer's Disease

**Figure 12:**
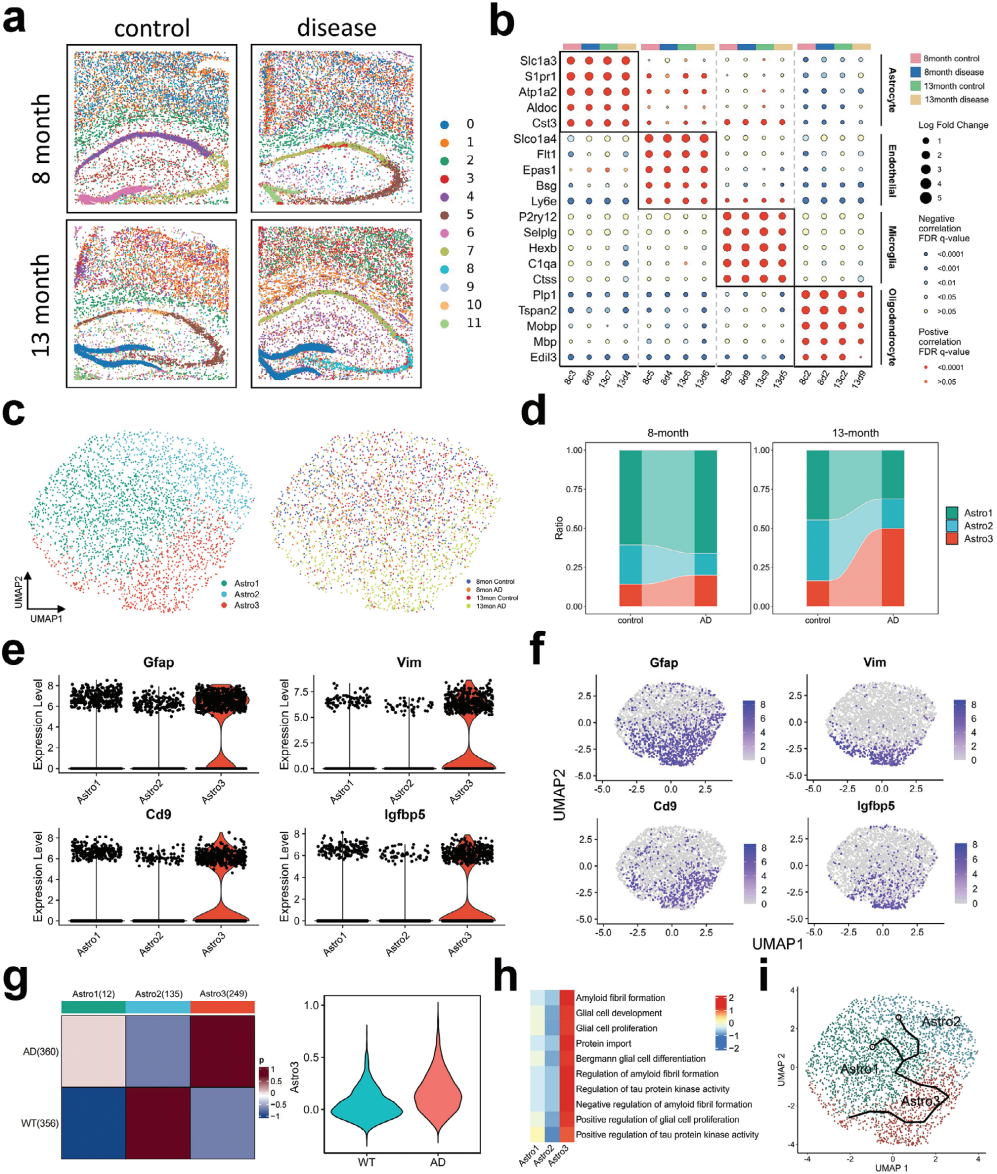
stDCL illustrates the underlying regulatory mechanisms in Alzheimer's disease. a) The spatial domains identified by stDCL on the STARmap PLUS dataset. b) The dot plot of marker gene expression levels in different cell populations identified by stDCL. c) The UMAP visualization of astrocyte subtypes. d) The comparison of different astrocyte subtypes proportions in AD and control samples at different times. e) The violin plot of expression levels of top marker genes in Astro3. f) The UMAP visualization of expression levels of top marker genes in Astro3. g) The MIA map of WT and AD astrocytes in sNuc‐seq and stDCL‐identified astrocyte subtypes in ST (left). The violin plot of expression levels of Astro3 gene in WT and AD astrocytes (right). h) The gene ontology enrichment analysis results of DAA‐like populations in astrocytes. i) The visualization of pseudotime trajectories of different astrocyte subtypes.

Alzheimer's disease (AD), a progressive neurodegenerative disorder, is marked by distinct pathologies in brain tissue, including reactive astrocyte changes. To illustrate the underlying regulatory mechanisms, we applied stDCL to the STARmap PLUS dataset,^[^ ^56^ ^]^ which comprises AD and control mouse brains from 8‐month and 13‐month specimens (**Figure** **12**a). stDCL identifies several cell clusters, including astrocytes, endothelial cells, microglia, oligodendrocytes, etc (Figure 12b and Figure S31, Supporting Information). We integrated all astrocytes identified by stDCL and performed subclustering analysis to obtain three distinct subpopulations Astro1, Astro2, and Astro3 (Figure 12c). Notably, the proportion of Astro3 was higher in AD than in controls, and Astro3 was significantly increased in AD from 8 to 13 months, suggesting a dynamic cellular response in AD progression (Figure 12d). Compared to other astrocyte subtypes, *Gfap*, *Vim*, *Cd9*, and *Igfbp5* were significantly upregulated in Astro3, which aligns with the disease‐associated astrocyte (DAA) phenotype reported in literature.^[^ ^57^, ^58^ ^]^ *Gfap* is primarily expressed in astrocytes and significantly elevated in the brains of Alzheimer's disease (AD) patients, where it correlates with gliosis and the presence of A β; plaques.^[^ ^59^, ^60^ ^]^ As a potential blood biomarker for AD,^[^ ^61^ ^]^ *Gfap* could aid in diagnosing patients and tracking disease progression and therapeutic efficacy.^[^ ^62^ ^]^ *Vim* is expressed in response to damage in brains, and its upregulation in Alzheimer's disease may play a role in neuronal repair following synaptic disruption and dendrite retraction,^[^ ^63^ ^]^ although its precise function in this context remains to be clarified. To further validate the association of Astro3 with the DAA‐like cell population, we introduced single nuclei RNA sequencing (sNuc‐seq) datasets of 10‐month‐old WT and AD (5xFAD) mouse brains (GSE143758),^[^ ^64^ ^]^ including 1179 WT astrocytes and 2079 disease‐associated astrocytes. We performed multimodal intersection analysis (MIA)^[^ ^64^ ^]^ using astrocytes from the sNuc‐seq dataset with astrocytes identified by stDCL in the STARmap PLUS dataset. MIA showed that these AD astrocytes were significantly enriched in the Astro3 subpopulation, WT astrocytes were predominantly enriched in the Astro2 subpopulation, and these cells were not significantly enriched in the Astro1 subpopulation (Figure 12g). In addition, gene expression of Astro3 was also more relevant to AD astrocytes compared to WT astrocytes. These results demonstrate the strong association of the Astro3 subpopulation with AD, affirming its alignment with the DAA‐like population and suggesting its proliferation within AD cells as the pathology advances.

Then, to further explore the AD‐related gene regulation in the Astro3 subpopulation, we performed GO enrichment analysis (Figure 12h) on the differentially expressed genes. It can be observed that differentially expressed genes in Astro3 were involved in glial cell development and proliferation, amyloid fibril formation, and protein import, all of which are relevant to pathological processes. In addition, these genes were mainly enriched in the regulation of tau protein kinase activity, the imbalance of tau kinase and phosphatase activity is believed to lead to tau hyperphosphorylation in the disease,^[^ ^65^ ^]^ which is a major pathological characteristic of AD. Additionally, we also performed pseudotime trajectory inference across the three astrocyte subtypes, unveiling that Astro1 and Astro2, which are not associated with disease, represent the initial state of astrocytes, and progressively merge into the branch of the DAA‐like subtype Astro3, which is consistent with the trend of disease progression (Figure 12i).

## 3 Discussion

Spatial transcriptomics enables the quantification of gene expression while simultaneously capturing spatial gene locations. The integration of gene expression and spatial information aids in the identification of spatial gene expression clusters, offering a pathway to elucidate spatial heterogeneity. Nevertheless, the effective integration of spatial data remains an ongoing challenge. Here, we have developed a graph‐based contrastive learning method called stDCL to identify spatial domains and uncover spatial heterogeneity. In particular, dual graph contrastive learning has been proposed to synergize both the space‐aware contrastive learning and cluster‐level feature contrastive learning for the single‐cell spatial dissections of tissues.

Specifically, stDCL accurately identifies spatial domains in the human dorsolateral prefrontal cortex tissue, outperforming eight other state‐of‐the‐art methods in spatial clustering performance. In particular, stDCL can identify spatial domains that closely match with human annotations consistently on the datasets generated from different spatial transcriptomics techniques, while maintaining stable performance across different age groups. Based on the latent embedding representation, stDCL can uncover important interpretable gene candidates in tissue and aggregate their spatial expression information. Additionally, we have demonstrated stDCL's effectiveness in reconstructing hierarchical structures and enhancing differential expression analysis on the STARmap dataset. Compared to STAGATE and GraphST, the imputation matrix generated by stDCL preserves the relative position between layers in the original space and accurately reconstructs the expression patterns of individual layer‐specific differential genes. Furthermore, stDCL successfully characterized the complicated molecular and spatial heterogeneity within the mouse brain at single‐cell resolution.

Notably, stDCL can unravel developmental patterns in the brain of mouse embryos. We applied stDCL to identify spatial domains in the E1S1 slice of E9.5 embryos. Interestingly, stDCL found a cluster of highly correlated regions concentrated within the brain area, elucidating gene regulation relevant to early stages of mouse brain development. In the downstream cluster analysis of additional slices, stDCL revealed the correlations between identified regions within the brain and spinal cord, highlighting the co‐enrichment of crucial axon guidance mechanisms involved in forming commissural circuits connecting these two regions. To provide a comprehensive analysis, we employed the E1S1 slice of E10.5 embryos for validation, confirming the significant pathways implicated in the formation of neuronal circuits.

Furthermore, stDCL can reveal the underlying regulatory mechanisms in Alzheimer's disease. We used stDCL to perform spatial clustering on Alzheimer's disease (AD) datasets, annotating astrocytes using marker genes. From the analysis of the astrocyte subgroup, it was found that the Astro3 subtype represented a higher proportion of diseased cells compared to control cells. Therefore, we further verified that subtype Astro3 was a DAA‐like population through the sNuc‐seq dataset and explored its AD‐related biological processes. In addition, we conducted pseudotime trajectory analysis to infer trends in astrocyte changes during disease progression.

In summary, stDCL is a versatile approach based on dual graph contrastive learning to jointly analyze spatial transcriptomics datasets across various regions, considering both gene expression and spatial distribution. In the complex spatial structure, stDCL demonstrates the ability to identify spatial domains, enhance the interpretability of genes, recover spatial hierarchy, elucidate spatial heterogeneity, unveil developmental patterns, and elucidate disease regulatory mechanisms. We will continue to advance and refine the capabilities of stDCL with open‐source availability and reproducibility in the future.

## 4 Experimental Section

### Data processing

In this study, stDCL performs spatial clustering by utilizing both the gene expression matrix and spatial location information derived from spatial transcriptomics data. To elucidate this methodology further, a logarithmic transformation was initially applied to the raw gene expression matrix and subsequently normalized it based on library size using the SCANPY software package.^[^ ^66^ ^]^ After normalization, the gene expression counts were further scaled to unit variance and zero mean. For 10x Genomics and Stereo‐seq datasets, the top 3000 highly variable genes as input were carefully selected. In the case of datasets with a smaller number of genes, such as STARmap, osmFISH, and MERFISH, it was opted to utilize all available genes as input without any additional selection process. This comprehensive approach ensured the suitability of this analysis for a wide range of spatial transcriptomic datasets.

### Transcriptomics Profile‐Based Spatial Graph Construction

A transcriptomics profile‐based spatial graph was developed to characterize the relationships between spots. In previous studies, most methodologies relied either on spatial information alone or on prior clustering outcomes to capture spot‐to‐spot relationships. However, this kind of graph constructed only through spatial location distance and pre‐trained information cannot effectively describe the relationship between spots, especially when they were spatially distant but belong to the same domain. To comprehensively characterize the relationships between spots, an undirected graph was designed that integrates both gene expression information and spatial information. Specifically, the Euclidean distance between each spot and other spots was calculated through their spatial location information and gene expression profiles, respectively. For spatial location information, the top *k*_1_ nearest spots were selected to each spot as its neighbors, while opting for the top *k*_2_ nearest spots for gene expression information. Then, connections (edges) between spots that share neighborhood status were established, yielding two adjacency matrices **A** ^*spatial*^ and **A** ^*gene*^ respectively, which is defined as follows:

(1) $$A_{ij}^{spatial}=\left\{\begin{array}{cc} 1, & j\in N_{k_{1}}\left(i\right)\\ 0, & j\notin N_{k_{1}}\left(i\right) \end{array}\right.\;A_{ij}^{gene}=\left\{\begin{array}{cc} 1, & j\in N_{k_{2}}\left(i\right)\\ 0, & j\notin N_{k_{2}}\left(i\right) \end{array}\right.$$

where $N_{k_{1}}\left(i\right)$ and $N_{k_{2}}\left(i\right)$ represent the neighborhood of the spot *i* for spatial information and gene expression information, respectively. Afterwards, the parameter α ∈ (0, 1) was introduced to adjust the weight between spots within **A** ^*gene*^. Its primary purpose is to establish a relationship framework where spatial location information takes precedence, is supplemented, and refined by gene expression information. Finally, **A** ^*spatial*^ and **A** ^*gene*^ were integrated to yield a neighborhood graph **A** as follows:

(2) $$A=\min\left(A^{spatial}+\alpha A^{gene},1\right)$$

Among them, the reason for limiting the weights between spots to 1 is to prevent over‐correction of gene expression information.

### Graph Convolutional Autoencoder

To better integrate and capture gene expression information and spatial location information, a graph convolutional autoencoder was employed to learn the latent embedding representation for spatial transcriptomics data. Specifically, both the encoder and the decoder utilize a single‐layer graph convolutional network (GCN), taking the normalized gene expression matrix **X** and the adjacency matrix **A** constructed from spatial information as input. Therefore, for spatial transcriptomics data, the latent embedding representation **Z** generated by the encoder and the reconstructed gene expression matrix **H** generated by the decoder can be defined as:

(3) $$Z=\sigma (D^{-\frac{1}{2}}AD^{-\frac{1}{2}}XW_{e})$$

(4) $$H=\sigma (D^{-\frac{1}{2}}AD^{-\frac{1}{2}}ZW_{d})$$

where σ(·) denotes the nonlinear activation function; **D** = *diag*{(**I** + **A**)**1**_*n*_} (*n* is the total number of spots) represents the degree matrix; **W**_*e*_ and **W**_*d*_ are the parameter matrices in the encoder and decoder, respectively. Further, an inner product decoder was designed to compute the reconstructed adjacency matrix $\hat{A}$, which is defined as follows:

(5) $$\hat{A}=\mathrm{sigmoid}\left(Z^{T}Z\right)$$

where sigmoid(*x*) = 1/(1 + *e* ^−*x*^) represents the nonlinear activation function. To jointly extract critical gene expression information and spatial location information, the reconstruction loss of the gene expression matrix was minimized and the adjacency matrix as follows:

(6) $$L_{rec}=\frac{1}{n}\sum_{i=1}^{n}\left(\alpha_{1}\left|\right|H_{i}-X_{i}{\left|\right|}^{2}+\alpha_{2}\left|\right|{\hat{A}}_{i}-A_{i}{\left|\right|}^{2}\right)$$

where *i* denotes the *i*‐th spot, α_1_ and α_2_ are weight coefficients.

### Space‐Aware Contrastive Learning

A space‐aware contrastive learning framework that leverages spatial information was proposed to ensure alignment between the spots in the embedding representations and their true spatial distribution. A space‐aware contrastive learning framework was proposed to improve the quality of the latent embedding representation through spatial information. First, data augmentation was performed to generate a corrupted gene expression matrix by adding Gaussian noise, which defined as:

(7) $$\hat{Z}=X⊙N_{g}$$

where **N**_*g*_ represents the random noise matrices obeying the Gaussian distribution, ⊙ is the Hadamard product. $\hat{X}$ was inserted into the encoder that shares parameters with **X** to generate the latent embedding representation $\hat{Z}$ under a different perspective as follows:

(8) $$\hat{Z}=\sigma \left(D^{-\frac{1}{2}}AD^{-\frac{1}{2}}\hat{X}W_{e}\right)$$

Contrastive learning endeavors to optimize the likeness between positive pairs while concurrently minimizing the resemblance among negative pairs. Inspired by this principle, a space‐aware contrastive learning approach tailored was proposed to spatial transcriptomics data. The space‐aware contrastive loss is established on a reasonable assumption that adjacent spots are similar and non‐adjacent spots are dissimilar. Therefore, the key idea is to enhance the similarity of spatially adjacent spots and weaken the similarity of non‐adjacent spots. Specifically, the cosine similarity matrix **S** of cross‐view latent embedding representations was calculated, which is defined as follows:

(9) $$S_{ij}=\frac{\left(Z_{i}\right){\left({\hat{Z}}_{j}\right)}^{T}}{\left|\right|Z_{i}\left|||\right|{\hat{Z}}_{j}\left|\right|}$$

where *S*_*ij*_ denotes the cosine similarity between the embedding representation of the *i*‐th spot in the first view and the embedding representation of the *j*‐th spot in the second view. To align the spot similarities with the spatial coordinate distribution, the mean square error (MSE) was employed between **S** and the adjacency matrix **A** as the contrastive loss, formulated as follows:

(10) $$L_{con}=\frac{1}{n^{2}}\sum_{i=1}^{n}\sum_{j=1}^{n}{\left(S_{ij}-A_{ij}\right)}^{2}$$

It is important to emphasize that during model training, as we minimized the contrastive loss, the similarity of spot pairs characterized by high weight in matrix **A** is increased while simultaneously observing a reduction in the similarity of pairs with lower weights. Obviously, this process clearly results in our space‐aware contrastive loss effectively bringing spatially adjacent spots into closer proximity and pushing non‐adjacent spots further apartx, ensuring that the embedding representation can effectively capture the spatial similarity inherent in the underlying organizational structure.

### Cluster‐Level Feature Contrastive Learning

Apart from adjusting the correlation of spots across views through spatial information, the correlation of features in the cluster‐level embedding representation was further optimized. The embedding representations of different views were equally divided into *k* groups (*k* is the number of clusters), calculated the average embedding within each group, and finally combined them to obtain the cluster‐level embedding representations. Specifically, the readout function was introduced $R\left(\cdot \right):R^{n\times d}\to R^{k\times d}$ (*d* is the latent embedding dimension) formulated as:

(11) $$R\left(Z\right)=\left[{\bar{Z}}_{1},{\bar{Z}}_{2},\text{…},{\bar{Z}}_{k}\right]$$

(12) $${\bar{Z}}_{i}=\frac{1}{|n_{i}|}\sum_{j\in n_{i}}Z_{j},\;i\in \left\{1,2,\text{…},k\right\}$$

where ${\bar{Z}}_{i}$ denotes the average embedding of group *i*; *n*_*i*_ represents the set of spots in group *i*; |*n*_*i*_| is the number of spots in group *i*. Likewise, still calculated the cosine similarity matrix $\tilde{S}\in R^{d\times d}$ of cross‐view cluster‐level embedding representations from the aspect of feature dimension, which is defined as follows:

(13) $${\tilde{S}}_{ij}=\frac{{\left(R{\left(Z\right)}_{i}\right)}^{T}\left(R{\left(\hat{Z}\right)}_{j}\right)}{{||R\left(Z\right)}_{i}||||R{\left(\hat{Z}\right)}_{j}\left|\right|}$$

where ${\tilde{S}}_{ij}$ denotes the cosine similarity between the average embedding of the *i*‐th dimension feature in all groups in the first view and the average embedding of the *j*‐th dimension feature in all groups in the second view. Representations of the same dimension feature across views were taken as positive pairs and maximize their similarity. Therefore, the MSE of $\tilde{S}$ and an identity matrix **I** ∈ **R** ^*d* × *d*^ were used as the cluster‐level contrastive loss, which is defined as follows:

(14) $$\begin{array}{cc} L_{clu} & =\frac{1}{d^{2}}\sum_{i=1}^{d}\sum_{j=1}^{d}{\left({\tilde{S}}_{ij}-I_{ij}\right)}^{2}\\ & =\frac{1}{d^{2}}\sum_{i=1}^{d}{\left({\tilde{S}}_{ii}-1\right)}^{2}+\frac{1}{d^{2}-d}\sum_{i=1}^{d}\sum_{j\neq i}{\left({\tilde{S}}_{ij}\right)}^{2} \end{array}$$

The cluster‐level feature contrastive learning strengthens the correlation between the same dimension features in two views and reduces the correlation between different dimension features. This can filter out the redundant information of latent embedding representation, preserve more discriminative features well, and improve the clustering performance of the model.

### Overall Loss Function

stDCL learns the latent embedding representation by minimizing the reconstruction loss $L_{rec}$, the space‐aware contrastive loss $L_{con}$, and the cluster‐level feature contrastive loss $L_{clu}$, which can be defined as:

(15) $$L=\gamma_{1}L_{rec}+\gamma_{2}L_{con}+\gamma_{3}L_{clu}$$

where γ_1_, γ_2_ and γ_3_ are weight coefficients assigned to each loss to control the balance of the overall loss function. γ_1_, γ_2_ and γ_3_ was set to [10, 0.5, 0.8] after parametric analysis. For model training, it was divided it into two parts: pre‐training and training, and both are optimized using the Adam optimizer. The learning rate and epoch for these two parts were 0.001, 0.005 and 500, 1000, respectively.

### Clustering and Refinement

To identify the spatial domains in our study, clustering techniques were employed on the latent embedding representations generated by stDCL. First, the dimension of the learned embedding representations of stDCL was reduced to 20 by PCA, and then used clustering methods to perform clustering. When the data is manually annotated, the mclust clustering algorithm was employed. For cases without manual annotation, the Leiden algorithm was used. To enhance the clustering performance, data augmentation were employed with two different Gaussian distributions and selected clustering results with relatively low Davies–Bouldin scores. Furthermore, stDCL incorporates a refinement process for the clustering results. Specifically, the neighborhood of each spot was defined as a circle with a radius of *r* (*r* is set to 50) and reassigned the spots to domains with the most common labels within their respective neighborhoods.

### Implementation Details and Comparisons with Baseline Methods

stDCL was implemented in Python, and its core model is built on the PyTorch framework, which is publicly available at https://github.com/Philyzh8/stDCL. To evaluate the performance of stDCL in spatial clustering, stDCL was compared with the following methods:

- *Seurat*. Seurat is a comprehensive software package written in R that facilitates the analysis of single cell data, including modules dedicated to processing spatial transcriptomics data. For data preprocessing, the default parameters in Seurat were utilized . To perform clustering, the appropriate resolution based on the desired number of clusters was determined and the “FindClusters” function was employed to assign cells to their respective clusters.
- *SpaGCN*. SpaGCN is a method based on graph convolutional networks that integrates three types of information including gene expression, spatial location, and histological images to identify spatial domains. The adjacency matrix in SpaGCN is constructed with parameters alpha' and beta' set to 1 and 49, respectively. During the training process, we set the learning rate to 0.05 and trained the model for 200 epochs.
- *stLearn*. stLearn uses the deep learning model to extract information from tissue morphology to normalize gene expression and improve spatial clustering. In this study, stLearn was employed for data normalization and spatial clustering, using the default parameter settings. The clustering method used in stLearn is the K‐means method.
- *BayesSpace*. BayesSpace is a Bayesian statistical clustering method that leverages information about spatial neighborhoods to enhance the resolution of spatial transcriptomics data and perform clustering analysis. The parameters were configured as follows: “d” was set to 15, “nrep” to 50 000, and “gamma” to 3.
- *SEDR*. SEDR (Spatial Embedding with Deep Representation) combines both an autoencoder and a variational autoencoder to generate embedding representations of transcripts and spatial information. During training, the number of epochs were set to 200, the learning rate to 0.01, and the dropout rate to 0.2.
- *CCST*. CCST is an unsupervised cell clustering method that uses GCNs to learn cell embedding representations from graphs derived from spatial transcriptomics data. For parameter settings, the default parameter settings were used in their code. The training process consisted of 5000 epochs, and the number of hidden layer nodes was set to 256. Additionally, the parameter “lambda_I” was set to 0.3.
- *STAGATE*. STAGATE is a graph attention autoencoder framework designed to learn a low‐dimensional latent embedding by combining spatial information and gene expression profiles. For data preprocessing, 3000 highly variable genes were specifically selected as input for analysis. The parameters “rad_cutoff” and “alpha” were set to 150 and 0, respectively.
- *GraphST*. GraphST is a graph self‐supervised comparative learning method that effectively utilizes spatial information and gene expression profiles for spatial clustering. The parameters set for model training were adopted from the recommended parameters in their code. For loss function weight assignment, the parameters “alpha” and “beta” were set to 10 and 1, respectively. The training process consisted of 600 epochs, with a learning rate of 0.001 and a dropout rate of 0.2.

### Benchmarking Metrics for stDCL Clustering

Three common external metrics were adopted to evaluate clustering performance, namely adjusted rand index (ARI), normalized mutual information (NMI), and homogeneity score (HS). ARI can measure the similarity between the cluster labels predicted by the algorithm and the real cluster labels. NMI quantifies the similarity of between two clusters by considering the mutual information between the predicted and true cluster labels, and normalizing it based on the entropy of the individual clusters. These two metrics can be defined as follows:

(16) ARI=∑ijnij2−∑iai2∑jbj2/n212∑iai2+∑jbj2−∑iai2∑jbj2/n2

(17) $$NMI=\frac{\sum_{ij}n_{ij}\log\frac{n_{ij}n}{a_{i}b_{j}}}{\sqrt{\sum_{i}a_{i}\log\frac{a_{i}}{n}\sum_{j}b_{j}\log\frac{b_{j}}{n}}}$$

where *n* is the total number of samples, *n*_*ij*_ is the number of samples belonging to both the *i*‐th cluster in the true label and the *j*‐th cluster in the predicted label, *a*_*i*_ and *b*_*j*_ are the total number of samples in the *i*‐th cluster in the true label and the *j*‐th cluster in the predicted label, respectively.

HS can measure the homogeneity of the clustering algorithm for sample division, which means that only samples of the same category are included in the cluster. The HS ranges from 0 to 1, where a higher value indicates a higher level of homogeneity in the clusters, which can be defined as:

(18) $$h=1-\frac{H(C|K)}{H(C)}$$

where *H*(*C*|*K*) is the conditional entropy of the true label partition under the predicted label partition condition.

In addition, the internal metric Davies‐Bouldin Index (DBI) was also used to improve the clustering performance of stDCL. DBI can calculate the average of the maximum ratio of the within‐cluster distance and the between‐cluster distance for each cluster, which can be formulated as:

(19) $$DBI=\frac{1}{n}\sum_{i=1}^{n}\max_{j\neq i}\left(\frac{\bar{S_{i}}+\bar{S_{j}}}{E_{ij}}\right)$$

where *n* is the total number of clusters, $\bar{S_{i}}$ denotes the average distance from the sample in the *i*‐th cluster to the cluster centroid, and *E*_*ij*_ represents the Euclidean distance between the *i*‐th cluster and the *j*‐th cluster. The lower the DBI, the better the clustering quality.

### Benchmarking Metrics for Stdcl Imputation

Four common metrics were adopted to evaluate the imputation performance of stDCL, namely Pearson correlation coefficient (PCC), Mean Squared Error (MSE), Spearman Rank Correlation Coefficient (SRCC), and Cosine Similarity (CS). These metrics evaluate the correlation and consistency of stDCL's imputation space with the original space. PCC was used to measure the linear correlation between the imputed and the true values. It captures the strength and direction of a linear relationship, with a value ranging from –1 to 1, where 1 indicates a perfect positive linear correlation and –1 indicates a perfect negative linear correlation, which can be defined as:

(20) $$PCC=\frac{\sum_{i=1}^{n}\left(x_{i}-\bar{x}\right)\left(y_{i}-\bar{y}\right)}{\sqrt{\sum_{i=1}^{n}{\left(x_{i}-\bar{x}\right)}^{2}}\sqrt{\sum_{i=1}^{n}{\left(y_{i}-\bar{y}\right)}^{2}}}$$

where *x*_*i*_ and *y*_*i*_ represent the true and imputed values, respectively, and $\bar{x}$ and $\bar{y}$ denote the mean of the true and imputed values.

MSE is used to measure the average squared difference between the true and imputed values. MSE penalizes larger deviations more heavily, which makes it sensitive to outliers. The lower the MSE, the better the imputation performance, which can be defined as:

(21) $$MSE=\frac{1}{n}\sum_{i=1}^{n}{\left(x_{i}-y_{i}\right)}^{2}$$

where *n* is the number of spots, and *x*_*i*_ and *y*_*i*_ are the true and imputed values, respectively.

SRCC measures the rank‐based correlation between the true and imputed values. Unlike PCC, SRCC is non‐parametric and assesses how well the relationship between the two variables can be described by a monotonic function, which can be formulated as:

(22) $$SRCC=\frac{\sum_{i=1}^{n}\left(r_{x_{i}}-\bar{r_{x}}\right)\left(r_{y_{i}}-\bar{r_{y}}\right)}{\sqrt{\sum_{i=1}^{n}{\left(r_{x_{i}}-\bar{r_{x}}\right)}^{2}}\sqrt{\sum_{i=1}^{n}{\left(r_{y_{i}}-\bar{r_{y}}\right)}^{2}}}$$

where $r_{x_{i}}$ and $r_{y_{i}}$ are the rank values of *x*_*i*_ and *y*_*i*_, respectively.

CS quantifies the similarity between two non‐zero vectors by measuring the cosine of the angle between them. It is particularly useful when comparing high‐dimensional data and ranges from 0 to 1, with 1 indicating perfect similarity, which can be defined as:

(23) $$CS=\frac{\sum_{i=1}^{n}x_{i}y_{i}}{\sqrt{\sum_{i=1}^{n}x_{i}^{2}}\sqrt{\sum_{i=1}^{n}y_{i}^{2}}}$$

where *x*_*i*_ and *y*_*i*_ are the true and imputed values.

### Differential Gene Expression Analysis

We performed differential gene expression (DE) analysis using the function “FindMarkers” in the R package Seurat (v4.0.2).^[^ ^29^ ^]^ The Wilcoxon rank sum test was applied to identify differentially expressed genes, and a Benjamini‐Hochberg correction was used to adjust the *p*‐values. Specifically, we selected the genes with adjusted *p*‐value ⩽ 0.05 as differentially expressed genes in the tested regions.

### Functional Enrichment

Gene Ontology (GO) and Kyoto Encyclopedia of Genes and Genomes (KEGG) enrichment analyses was performed on the differentially expressed genes using the R package ClusterProfiler (v3.18.1).^[^ ^67^ ^]^ The function “enrichGO” and “enrichKEGG” were applied to GO analysis and KEGG analysis. In both analyses, the parameters were set as follows: “pAdjustMethod = BH” and “pvalueCutoff = 0.05”. Subsequently, the results of the GO analysis were visually presented using the “heatplot” function from the R package enrichplot (v1.14.2). These analyses provided valuable insights into the enriched biological processes and pathways associated with the differentially expressed genes.

### PPI Network

STRING^[^ ^46^ ^]^ was utilized to generate a protein–protein interaction (PPI) network for the up‐regulated differentially expressed genes in the brain regions of mouse embryos. The PPI network was constructed using interactions with scores greater than 0.4. To visualize the network, Cytoscape was employed,^[^ ^68^ ^]^ which allowed us to gain a visual representation of the network and explore its underlying structure. Furthermore, MCODE^[^ ^47^ ^]^was adopted to identify significant coherent modules within the PPI network and showed the highest scoring modules.

### Trajectory Inference

The R package Slingshot (v2.2.1)^[^ ^69^ ^]^ was used for trajectory inference. Slingshot is a powerful tool designed to infer the structure and order of cell lineage differentiation, which consists of two main stages: inference of lineage structure, and inference of pseudotemporal changes in cells of each lineage. In this analysis, Slingshot was utilized on the latent representations obtained from stDCL. The predicted cluster labels were used as input for the “slingshot” function in Slingshot. This allowed us to leverage the learned latent representations to infer the trajectory and pseudotime information for the cells. Following trajectory inference, Slingshot assigned a pseudo‐time value to each location. In addition, Slingshot needed to set a domain as the starting domain in trajectory inference, and the White matter was set as the starting domain when analyzing the osmFISH dataset. This choice determined the initial reference point from which the trajectory was inferred, aiding in the interpretation of the differentiation process in the context of the specific dataset. Moreover, R package Monocle3 (v1.3.6) (http://cole‐trapnell‐lab.github.io/monocle‐release/monocle3) was used to infer pseudotime trajectories of astrocyte subtypes in the STARmap PLUS data. Monocle3 used the “learn_graph” function to learn principal graph in the UMAP space. Pseudotime was computed by calculating the geodesic distance from the specified root node to the cell node along the principal graph, and adopted the function “plot_cells” to visualize the trajectory inference.

### Gene Set Variation Analysis (GSVA)

Gene set variation analysis were performed using the R package GSVA (v1.38.2). In the analysis, the “gsva” function from the GSVA package was utilized. The GSVA enrichment scores were estimated using the “method = ssgsea” parameter setting. This method, based on the Single Sample Gene Set Enrichment Analysis (ssGSEA) approach, allowed us to evaluate the activity or enrichment levels of gene sets or pathways in each sample. The related pathway enrichment was used to assess the activation of WikiPathways pathways, which is available in the MSigDB database.^[^ ^70^ ^]^

## Conflict of Interest

The authors declare no conflict of interest.

## Supporting information

Supporting Information

## Data Availability

The data that support the findings of this study are openly available in Accurate Spatial Heterogeneity Dissection and Gene Regulation Interpretation for Spatial Transcriptomics using Dual Graph Contrastive Learning at https://doi.org/10.5281/zenodo.10968451.
