# Supplementary material for: Accurate Spatial Heterogeneity Dissection and Gene Regulation Interpretation for Spatial Transcriptomics using Dual Graph Contrastive Learning
Source: Adv Sci (Weinh). 2024 Nov 28;12(3):2410081. doi: 10.1002/advs.202410081 (PMC11744562; doi:10.1002/advs.202410081)
Supplement: Supplementary file 1 — Supporting Information [file ADVS-12-2410081-s001.pdf]

Supplementary Information “Accurate Spatial Heterogeneity Dissection and Gene Regulation Interpretation for Spatial Transcriptomics using Dual Graph Contrastive Learning''

Zhuohan Yu[1], Yuning Yang[2], Xingjian Chen[3], Ka-Chun Wong[4], Zhaolei Zhang[2], Yuming Zhao[5*], Xiangtao Li[1,4*]

[1]School of Artificial Intelligence, Jilin University, Jilin, China

[2]Terrence Donnelly Centre for Cellular and Biomolecular Research, University of Toronto, Toronto, ON, M5S 3E1, Canada

[3]Cutaneous Biology Research Center, Massachusetts General Hospital, Harvard Medical School, Boston, MA, USA

[4]Department of Computer Science, City University of Hong Kong, Hong Kong SAR

[5]College of Computer and Control Engineering, Northeast Forestry University, Harbin, China

DRAFT

Supplementary Note 1: Comparison of spatial domains identified by stDCL and other methods on DLPFC.

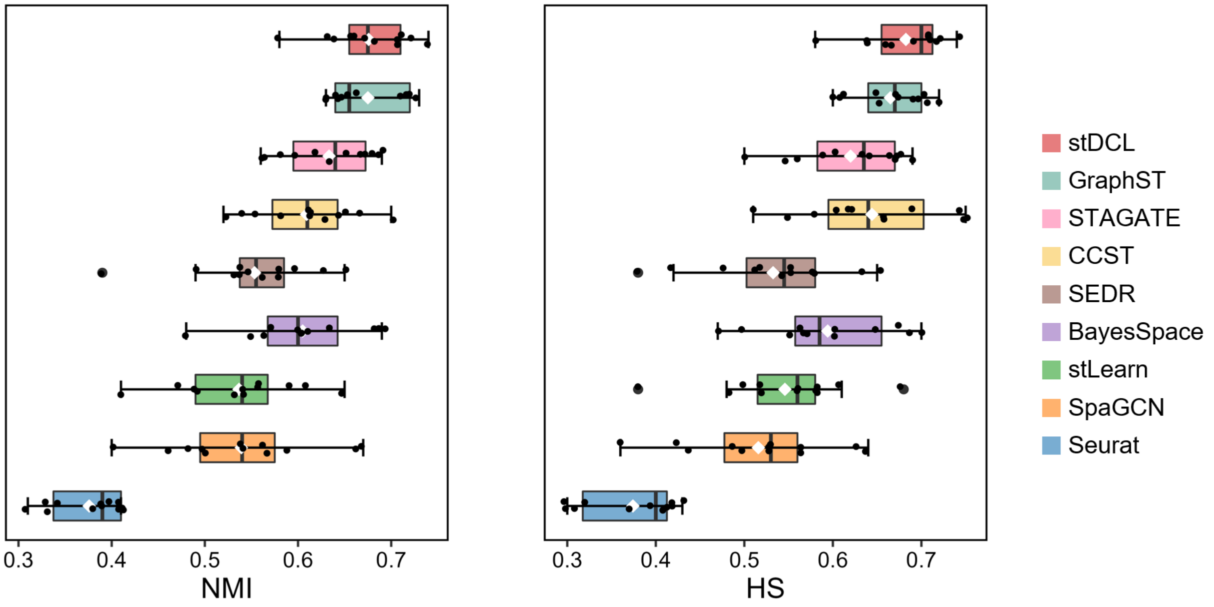

Figure S1. Boxplot of clustering accuracy in all 12 DLPFC slices dataset in terms of NMI and HS scores for 9 methods (n = 12 in each group; center line, median; box limits, upper and lower quartiles; whiskers, 1.5× interquartile range).

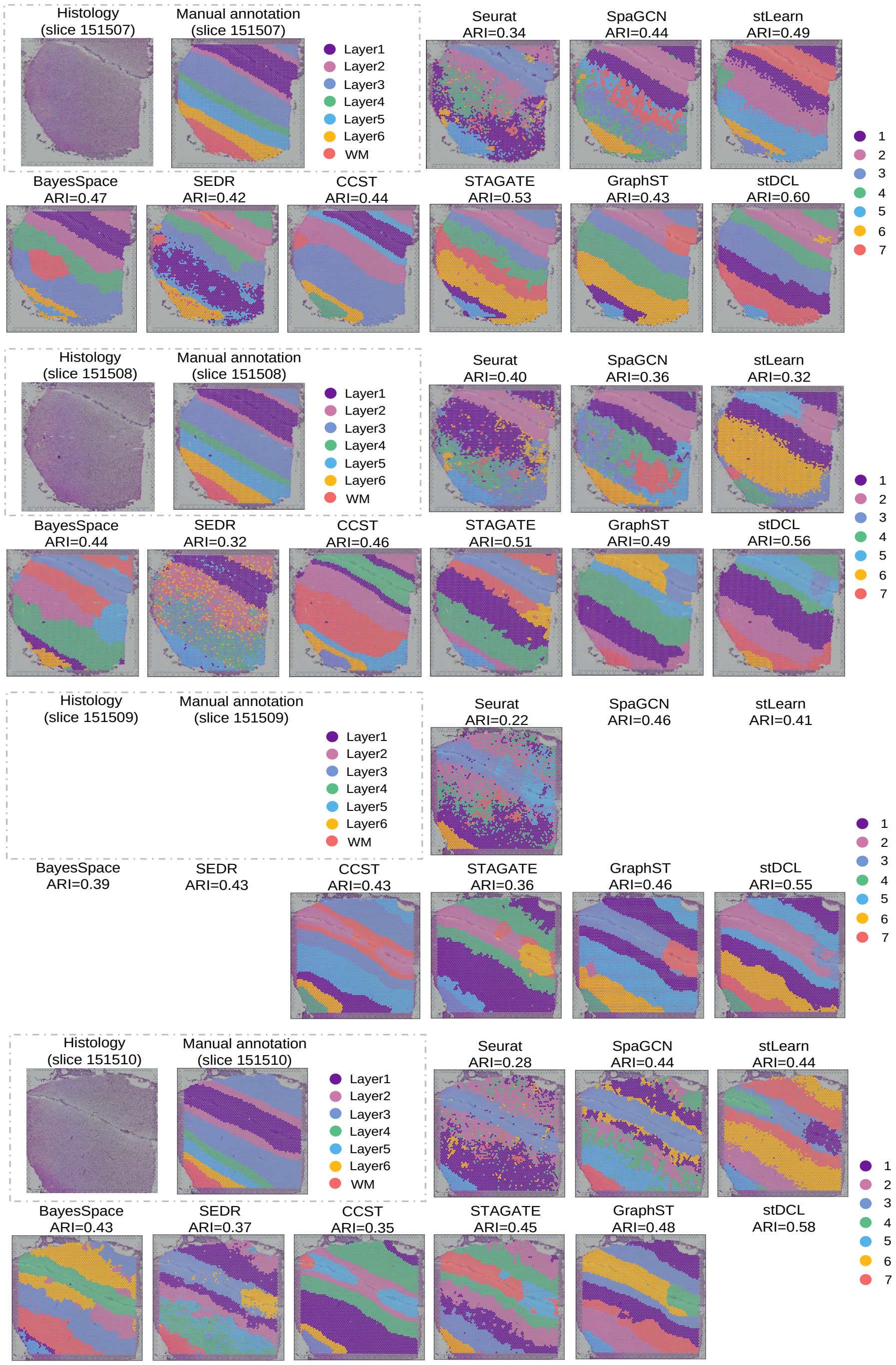

Figure S2. Manually annotated layer structure and spatial domains detected by Seurat, SpaGCN, stLearn, BayesSpace, SEDR, CCST, STAGATE, GraphST, and stDCL on slice 151507, 151508, 151509, and 151510.
